# Signaling pathways mediating the induction of preharvest fruit drop in litchi

**DOI:** 10.3389/fpls.2024.1474657

**Published:** 2024-12-09

**Authors:** Jun Wang, Wuqiang Ma, Fei Wang, Zidi He, Xiangyang Ye, Jiahui Deng, Minglei Zhao, Jianguo Li

**Affiliations:** ^1^ State Key Laboratory for Conservation and Utilization of Subtropical Agro-Bioresources, College of Horticulture, South China Agricultural University, Guangzhou, China; ^2^ Ministry of Agriculture and Rural Affairs Key Laboratory of South China Horticultural Crop Biology and Germplasm Enhancement, College of Horticulture, South China Agricultural University, Guangzhou, China; ^3^ School of Tropical Agriculture and Forestry (School of Agricultural and Rural Affairs, School of Rural Revitalization), Hainan University, Danzhou, China; ^4^ Sanya Nanfan Research Institute, Hainan University, Sanya, China

**Keywords:** Litchi *chinensis Sonn.*, preharvest fruit drop, hormone, abscission signals, transcriptome

## Abstract

Certain litchi varieties, such as “Nuomici”, are highly susceptible to preharvest fruit drop, which leads to significant losses in fruit yield and economic value. However, the precise molecular mechanisms underlying this issue are not yet fully understood. In this study, we aimed to elucidate the signaling pathways that facilitate preharvest fruit drop in litchi, using “Nuomici” and “Huaizhi” cultivars as examples, which demonstrate high and low preharvest fruit drop rates, respectively. Our findings revealed that “Nuomici” experienced a substantial preharvest fruit drop, with a cumulative rate of 41.68%, significantly higher than the 1.44% observed in “Huaizhi”. Cellulase activity assays showed a significant increase in cellulase activity in the abscission zone of “Nuomici”, which coincided with the occurrence of preharvest fruit drop, in contrast to the relatively low levels in “Huaizhi”. Phytohormone assays indicated lower indole-3-acetic acid content in the pericarp, aril, and seeds of “Nuomici” during the preharvest stage compared to “Huaizhi”, coupled with higher abscisic acid levels in the seeds of “Nuomici”. Furthermore, transcriptomic analysis identified 180, 282, 655, and 241 differentially expressed genes (DEGs) in the pericarp, aril, seed, and abscission zone, respectively, between the two cultivars during preharvest fruit drop. These DEGs are intricately involved in the generation and transmission of abscission signals from fruit tissues, encompassing *PIN*, *PIN-LIKES*, *LAX*, and *SAUR* genes related to polar auxin transport, ethylene diffusion, as well as perceiving these signals and activating the abscission process within the abscission zone. This includes *ACO* and *ILR* genes involved in hormone biosynthesis and signal transduction, regulation by WRKY, NAC, and bHLH transcription factors, AAO genes involved in response to reactive oxygen species, and EXP, EG, and PG genes involved in cell wall degradation in the abscission zone. Based on these comprehensive findings, we propose a model for preharvest fruit drop triggered by a series of molecular events in litchi, providing valuable insights into the complex mechanisms governing this phenomenon.

## 1 Introduction

Preharvest fruit drop is a phenomenon characterized by the premature falling of fruits from the tree before they are ready for harvest due to either developmental or adverse environmental cues (Arseneault and Cline, 2016; Zhao and Li, 2020). These abscission cues typically originate from the mother plant or from the fruits that are about to be shed. These signals are transmitted to the abscission zone (AZ), a specialized region of the plant where the actual separation of the fruit occurs. The reception of abscission signals triggers a cascade of coordinated events in the abscission zone, including signal perception, cell separation, and protective layer formation, ultimately leading to fruit shedding (Zhao et al., 2024). However, to the best of our knowledge, current preharvest fruit drop research focuses separately on abscission signal generation in fruits and signal perception in the AZ, lacking a systematic investigation of their interconnections, thus limiting a comprehensive understanding of the phenomenon.

As a crucial component of abscission cues, phytohormones within fruits play a pivotal role in plant organs shedding. The phytohormones indole-3-acetic acid (IAA) and ethylene have long been known to act in antagonistic roles in regulating the abscission processes (Meir et al., 2010). As early as 1955, Addicott and Lynch (1955) proposed the auxin gradient theory, suggesting that it is not the absolute content of auxin but the relative concentration, i.e., the IAA concentration gradient on either side of the abscission layer, that governs the regulation of organ abscission. IAA in fruits, as the primary source of auxin in fruit AZ, plays a key role in maintaining polar auxin transport (PAT) in the AZ and preventing immature fruit drop. Studies on sweet orange and apple have shown that enhancing the content of IAA and IAA-like compounds within the fruit can effectively reduce preharvest fruit drop, indicating that IAA in fruit serves as a positive regulatory signal for controlling preharvest fruit drop (Agustí et al., 2009; Anthony and Coggins, 1999). These studies suggest that the IAA present in the fruit may positively regulate the PAT mechanism within the AZ, thereby negatively modulating the occurrence of preharvest fruit drop. As a gaseous hormone, ethylene in the fruit is more likely to diffuse from the fruit to the AZ, inducing cell wall degradation and leading to fruit abscission (Botton and Ruperti, 2019). The exogenous application of the ethylene-releasing compound before harvest has consistently been demonstrated to reduce fruit removal force in “Bing” sweet cherry (*Prunus avium* L.) (Elfving et al., 2009). Preharvest application of ethylene synthesis inhibitors such as AVG (Aminoethoxyvinylglycine) or 1-MCP (1-methylcyclopropene) in apple can reduce the ethylene concentration in the fruit and mitigate preharvest drop (Dal Cin et al., 2008; Yildiz et al., 2012; Yuan and Carbaugh, 2007; Yuan and Li, 2008). However, ethylene in the fruit may not be the sole upstream factors that inducing preharvest fruit drop. Sun et al. (2009) investigated preharvest fruit drop in 114 apple cultivars and found that in some cultivars with preharvest drop, the occurrence of preharvest fruit drop prior to substantial production of ethylene in the fruit. Collectively, cumulative evidence suggests ethylene as a key signaling molecule in preharvest fruit drop regulation, albeit likely acting in concert with other factors in this complex physiological process.

In addition to the well-characterized roles of auxin and ethylene, the phytohormones abscisic acid (ABA), cytokinin and polyamines have also been identified as import complex regulators of preharvest fruit drop processes (Wu et al., 2024). In pummelo, it has been found that preharvest fruit drop is associated with an increase in ABA content within the fruit (Nartvaranant, 2015). However, the specific mechanisms by which ABA regulates organ shedding processes remain elusive. Cytokinin, as a hormone that promotes cell division and differentiation, has also been implicated in the suppression of preharvest fruit drop. Spraying 100 mg/L of the cytokinin analogue forchlorfenuron at full bloom or 2 weeks after full bloom could retard preharvest fruit drop of cv. *Early McIntosh* in Massachusetts for approximately 7 days (Curry and Greene, 1993). Similarly, sprayed 200 mg/L of benzyladenine (a cytokinin analogue) on *Macadamia* after flowering, also delaying the abscission of immature fruits (Trueman, 2010). Spermidine biosynthesis inhibitors, such as cyclohexylamine and methylglyoxal bis-guanylhydrazone, can effectively induce the abscission of mature olive fruits (Parra-Lobato and Gomez-Jimenez, 2011). However, the exact primary roles of these three hormones (ABA, cytokinin and polyamines) in preharvest fruit drop process remain unclear and require further research to elucidate their specific functions. Collectively, these evidences indicates that the regulation of preharvest fruit drop involves the complex interplay of multiple hormonal signals.

During preharvest fruit drop, the abscission process is activated in the AZ upon receiving signals from the fruit. The intricate regulatory mechanisms governing preharvest fruit drop in the AZ have been the focus of extensive research across various species. Gil-Amado and Gomez-Jimenez (2013) identified two distinct stages of preharvest olive drop: one at 154 days after flowering (DAF) prior to abscission and another at 217 DAF during active abscission. Through comparative transcriptional profiling of AZ samples using RNA-seq, they revealed that AZ cell separation entails the remodeling of membrane sterol/sphingolipid composition, signaling protein functions, cell wall modifications, hormone signal transduction, and transcriptional regulation. Corbacho et al. (2013) compared the transcriptomes of the AZ at three stages and found that the genes activated during preharvest drop in melon are related to cell wall metabolism, endomembrane transport, protein phosphorylation, plant hormone biosynthesis and signal transduction, and ion flux, involving transcription factors such as AP2/ERF, Aux/IAA, ZIP, HB, ZF, and WRKY. Preharvest fruit drop induced by externally applied hormones or pests/diseases also involves processes related to hormone biosynthesis and signal transduction, transcriptional regulation, and cell wall modifications. Cheng et al. (2015) reported that the induction of preharvest fruit drop in citrus by exogenous ethylene treatment was accompanied by the activation of the ethylene signaling pathway and the downregulation of genes involved in carbohydrate/water transport and the synthesis of growth-promoting hormones within the pedicel AZ. Transcriptomic analysis of the calyx AZ revealed upregulated expression of genes related to ethylene, jasmonic acid, and ABA synthesis and signaling in HLB (Huanglongbing) affected abscised fruits compared to healthy fruits, suggesting these hormones involvement in biotic stress-induced preharvest fruit drop (Zhao et al., 2019). Overall, both natural and environmentally induced preharvest fruit drop tend to induce extensive transcriptional reprogramming within the AZ in response to the abscission process. However, the lack of concurrent fruit and AZ transcriptomics during preharvest fruit drop has limited our understanding of the regulatory mechanisms bridging abscission signaling in the fruit to its execution in the AZ.

As an important tropical and subtropical fruit crop, litchi (*Litchi chinensis* Sonn.) is highly valued in the market for its unique flavor and high nutritional content. However, litchi undergoes four to five distinct physiological fruit drop events, with the preharvest stage suffering a particularly high 20-50% loss of fruit (Zhao and Li, 2020). Therefore, to elucidate the mechanisms underlying litchi preharvest fruit drop and to reduce its impact on yield, research efforts have been focused on two crucial organs: the abscising fruit and the abscission zone. With regard to litchi fruit, current research primarily explores the relationship between the hormonal balance within the fruit and the occurrence of preharvest fruit drop. Xiang et al. (1995) investigated the relationship between physiological fruit drop and hormones in the “Nuomici” litchi variety and observed that the peak of fruit abscission regularly occurred one week following the peak of ABA in the ovary. Qiu et al. (1998) compared the contents of IAA, cytokinin, and ABA in the pericarp of three types of litchi fruit at different stages of fruit drop, suggesting that an increased ratio of (IAA+GAs+CTK)/ABA may potentially reduce preharvest fruit drop in litchi. These evidences indicates that fruit-localized ABA might enhance preharvest fruit drop, while IAA and cytokinin within the fruit could inhibit this process. Similar to the fruit, research on the AZ and preharvest fruit drop has also been primarily focused on hormonal associations. Chen et al. (2018) found that the expression of *LcNCED2* in the AZ correlated with the trend of preharvest fruit drop in “Wuheli” variety, indicating its likely involvement in preharvest fruit drop. Molecular studies on the AZ have uncovered a potential link between abscisic acid biosynthesis and preharvest fruit drop. This connection has only been observed within the AZ. Nevertheless, the exact role of ABA in this process remains unclear. Additional investigation is required to clarify its specific function in preharvest fruit abscission. Overall, physiological and molecular studies have highlighted the pivotal role of hormones in litchi preharvest fruit drop, there remains a significant gap in understanding the comprehensive molecular mechanisms underlying this phenomenon.

In this study, we aimed to elucidate the signaling pathways responsible for the preharvest fruit drop phenomenon in litchi. To achieve this, we compared two litchi cultivars with contrasting susceptibilities to preharvest fruit drop: “Nuomici”, which exhibits high preharvest fruit drop, and “Huaizhi”, which exhibits low preharvest fruit drop. Ultra-performance liquid chromatography-electrospray ionization-tandem mass spectrometry (UPLC-ESI-MS/MS) and spectrophotometry were employed to quantify the concentrations of IAA and ABA across various fruit tissues and the cellulase activity in AZ, respectively. Concurrently, differential expressed genes (DEGs) were identified through comprehensive transcriptome data analysis across various fruit tissues and the AZ. Subsequently, by integrating the data from the quantification of plant hormones, enzyme activity assays, and transcriptome sequencing, we preliminarily unveiled the molecular mechanism by which the fruit “remotely controls” the AZ to promote abscission in the context of litchi preharvest fruit drop.

## 2 Materials and methods

### 2.1 Sample collection and data statistics

Three 28-year-old “Huaizhi” litchi trees and three 28-year-old “Nuomici” litchi trees with similar initial fruit bearing were randomly selected at the Xili orchard in Shengzhen, China. Forty-seven days post anthesis (DPA), twenty fruit-bearing shoots positioned in different directions on each tree were selected and tagged. These tagged shoots were used to monitor fruit abscission rate, while the untagged shoots were allocated for sample collection. Samples of the pericarp, aril, seed and AZ were collected at 47, 55, 62 and 69 DPA, respectively. Immediately following collection, all samples were frozen in liquid nitrogen and stored at -80°C for future analysis. Each tree was considered an independent biological replicate.

Relative Fruit Abscission Rate (RFAR) and Cumulative Fruit Abscission Rate (CFAR) was calculated using the following formula (Huang, 2002; Li et al., 2015b):


$$\text{RFAR }\left(\text{Relative Abscission Rate}\right)=100*\left(X_{t}-\;X_{t}-1\right)\;/\;\left(X_{t-1}*d\right)$$

$$\text{CFAR }\left(\text{Cumulative Fruit Abscission Rate}\right)=100*\left(X_{0}-X_{t}\right)/X_{0}$$

where, “X” represents the abscission records taken at any time “t” and “t-1”, “d” interval (in days) elapsing between “t-1” and “t”, “X_0_” represents the initial number.

### 2.2 Assays for the ABA and IAA content

The concentrations of ABA and IAA in 300 mg samples of litchi fruit were quantified using a validated UPLC-ESI-MS/MS method, as described by Xu et al. (2022). In brief, the extraction of the plant hormones was performed using chilled 80% acetone, followed by C^18^ solid-phase purification, and gradient elution separation using UPLC. Isotope-labeled internal standards (D^6^-ABA and ^13^C-IAA) were employed for accurate quantification. The contents of ABA and IAA in the samples were calculated according to the following formula:

Content = (Analytic ion peak area × IS product ion peak area)*/*(IS concentration × fresh weight of sample).

### 2.3 Cellulase enzymes activity measurements

300 mg AZ samples were collected at 47, 55, 62 and 69 DPA, respectively, and were homogenized in 3 ml extract by ice bath immediately. The homogenate was then centrifuged at 8,000×g at 4°C for 10 min. The supernatant obtained was used for enzyme determination. The cellulolytic enzyme activity was quantified using the Cellulase Assay Kit (Cat #BC1580, Solarbio, Beijing, China).

### 2.4 cDNA library construction and illumina sequencing

A total of 100 mg of frozen pericarp, aril, seed or AZ samples were powdered under liquid nitrogen and total RNA was extracted using RNAprep Pure Plant Kit (Cat #DP432, TIANGEN Inc., Beijing, China) according to the manufacturer’s instructions. Subsequently, each RNA sample was treated with DNase digestion (Cat #2270B, TaKaRa, Dalian, China) to eliminate any residual DNA. The RNA concentration was determined via spectrophotometry using a BioPhotometer plus instrument (Eppendoff, Germany), and the integrity of the RNA was verified by analyzing 1.2% denaturing agarose gels. The RNA Integrity Number (RIN) values of the samples were assessed using an Agilent 2100 Bioanalyzer (Agilent Technologies, Santa Clara, CA, USA), with values greater than 8.0 considered acceptable. The library construction and RNA-Seq were performed by the Biomarker Biotechnology Corporation (Beijing, China). The cDNA library preparation followed the standard Illumina sample protocol. The purified libraries were sequenced on an IlluminaHiseqTM2500 (Illumina, Inc., USA).

### 2.5 Sequence data analysis

The data were cleaned by removing adapter sequences, poly-N regions, and low-quality reads from the raw data. The quality of the cleaned data was assessed by calculating the Q30 score and GC and GC content. The clean paired end reads were then mapped to the reference genome using Hisat software (Pertea et al., 2016). Subsequently, the read counts and normalized gene expression levels were calculated with FPKM (Fragments Per Kilobase Million) values by Stringtie tool (Pertea et al., 2015). Finally, all detected genes were annotated against the NR (Nucleotide Resource), Swiss-Prot, COG (Clusters of Orthologous Groups), and KEGG (Kyoto Encyclopedia of Genes and Genomes) databases.

### 2.6 Screening of DEGs related to preharvest fruit drop

Using AZ tissue as an example, differentially expressed genes (DEG) associated with preharvest fruit drop were identified by a comparative analysis of gene expression profiles. This involved contrasting the profiles of samples from the “Nuomici” harvested at 55, 62, and 69 DPA with those collected at 47 DPA, as well as with concurrent samples from the “Huaizhi”. Genes that satisfied a statistical threshold of False Discovery Rate (FDR) ≤ 0.05 and a fold change (FC) absolute value ∣log_2_(FC)∣>1 in both the “Nuomici” at different DPA comparisons and the “Huaizhi” comparison were selected, which resulting in three distinct sets of AZ-specific DEGs at 55, 62, and 69 DPA, respectively. This approach was replicated for the pericarp, aril and seed tissues to compile a comprehensive dataset of DEGs associated with preharvest fruit drop across the various tissues and developmental stages. Furthermore, these identified DEGs within each tissue were subjected to k-means clustering analysis using the scikit-learn package in Python to identify co-expression patterns.

### 2.7 Co-expression network analysis

Weighted gene co-expression network analysis was conducted using WGCNA package (1.70) to identify gene co-expression patterns based on the normalized count matrix (Pei et al., 2017). A signed network was constructed using a soft-thresholding power selected to achieve approximate scale-free topology. Pearson correlation coefficients were calculated between gene pairs to measure their co-expression strength. The significant correlations between differentially expressed genes (threshold *p*< 0.01) were extracted and visualized using Cytoscape software (3.8.2) to construct the co-expression network (Saito et al., 2012).

### 2.8 Quantitative RT-PCR validation

RNA was reverse-transcribed into cDNA by TransScript^®^ One-Step gDNA Removal and cDNA Synthesis SuperMix (Cat #AT311-02, TransGen Biotech, Beijing, China) with gDNA Remover, Q-PCR was performed on the LightCycler 480II (Roche, Basel, Sweden) using SYBR Green Realtime PCR Master Mix (Cat #QPK-201, TOYOBO CO, LTD, Japan) as the readout. The running parameters of PCR amplifications included the following condition: 95°C for denaturation at 30 s, followed by 40 cycles of denaturation at 95°C for 5 s, annealing at 60°C for 30 s and extension at 72°C for 30 s. Then, PCR products were analyzed by the melting curve to confirm the specificity of amplification. Primers were designed using Primer5. In this study, *LcActin* -normalized expressions are presented (Zhong et al., 2011). Relative gene expression levels were calculated using 2^-△△CT^ method with three independent biological replicates.

### 2.9 Statistical analysis

Statistical analysis of physiological parameters was conducted using Student’s *t*-test with three biological replicates (*p*< 0.05). For transcriptome data analysis, we first determined the optimal number of clusters through silhouette analysis (cluster range: 2-15), followed by K-means clustering implementation using sklearn (1.4.0) package in Python to classify genes with similar expression patterns. Functional annotation of differentially expressed genes was performed using ClusterProfiler (4.10.0) package in R (Wu et al., 2021). Gene Ontology (GO) terms, including biological process, molecular function, and cellular component categories, and Kyoto Encyclopedia of Genes and Genomes (KEGG) pathways were analyzed based on eggNOG annotations. The significance of enrichment was determined using hypergeometric distribution test (*p*< 0.05). GO hierarchical analysis was visualized using TopGO (2.54.0) package in R.

## 3 Results

### 3.1 Dynamics of preharvest fruit drop between “Nuomici” and “Huaizhi”

**Figure 1:**
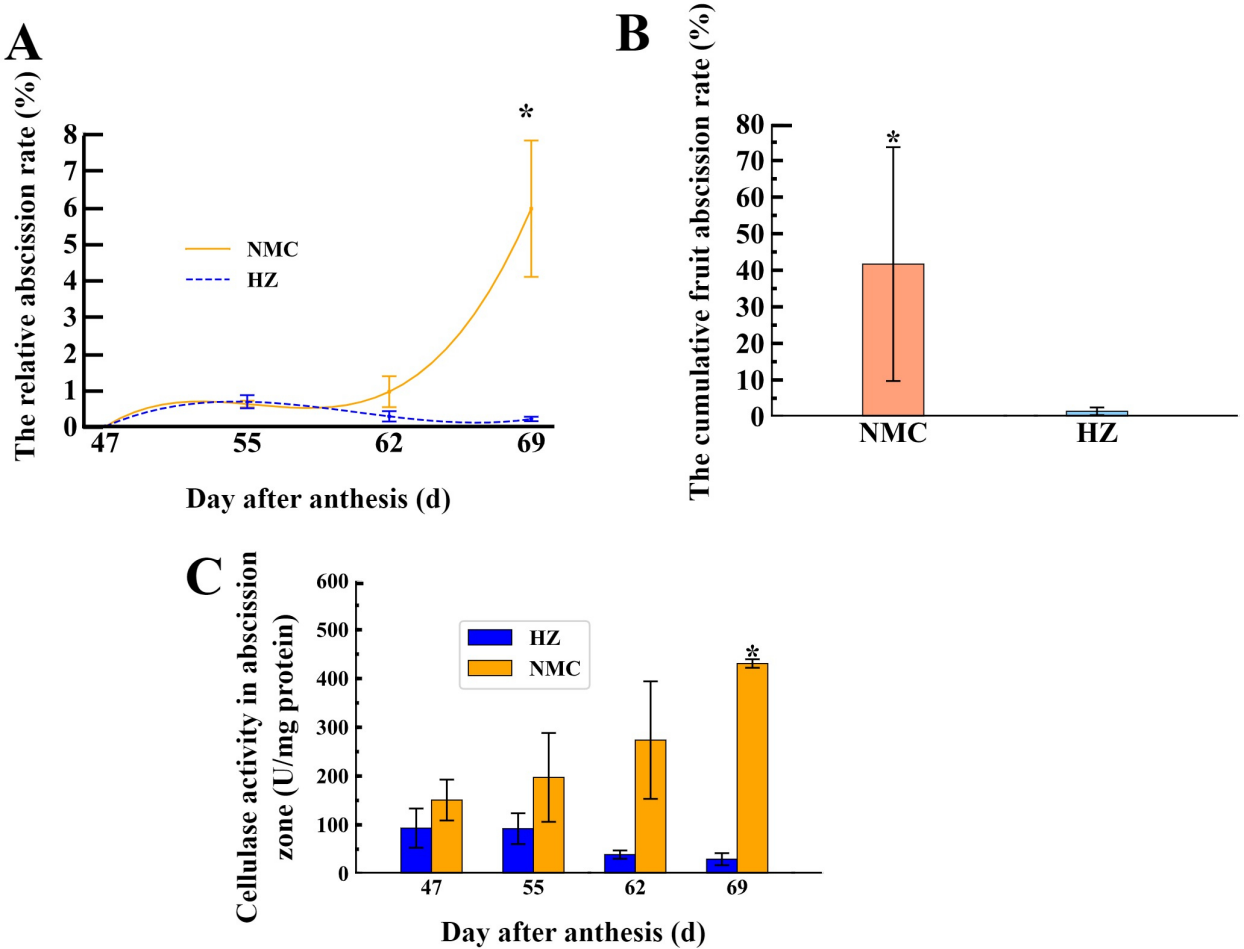
Dynamics of preharvest fruit drop **(A, B)** and the relative activity of cellulase in the fruit abscission zone (AZ) **(C)** between “Nuomici” (NMC) and “Huaizhi” (HZ) litchi cultivars. Vertical bars represent the standard error of three biological replicates, significant differences at 0.05 level are indicated with one asterisk (*) according to student t-test.

To investigate the dynamics of preharvest fruit drop in the “Nuomici” and “Huaizhi” cultivars, this study tracked the daily relative fruit drop rate from 47 to 69 DPA. As shown in **Figure 1A**, the peak of preharvest fruit drop in “Nuomici” occurred at 69 DPA, with a daily relative fruit drop rate of 5.95%. In contrast, “Huaizhi” exhibited no noticeable peak in preharvest fruit drop. Furthermore, the cumulative fruit drop rate of “Nuomici” was as high as 41.68% during preharvest fruit drop, which was significantly higher than the 1.44% observed in “Huaizhi” (**Figure 1B**). These findings suggest that “Nuomici” suffered from severe preharvest fruit drop.

To determine whether preharvest fruit drop is associated with cell wall degradation in the AZ, we conducted enzyme activity assays to assess the cellulase activity differences in the AZ between the two varieties during preharvest fruit drop. As illustrated in **Figure 1C**, the cellulase activity in the AZ of “Nuomici” increased gradually with the occurrence of preharvest fruit drop, reaching a peak of 430.74 U/mg protein at 69 DPA, which was significantly higher than the 29.58 U/mg protein observed in “Huaizhi” at the same timepoint. Conversely, the cellulase activity in the AZ of “Huaizhi” exhibited a consistent decline, with its highest activity at 47 DPA, reaching 93.22 U/mg protein, which was lower than the minimum value observed in “Nuomici”. In summary, the patterns of cellulase activity observed in the AZ of both cultivars suggest enhanced degradation of cellulose in “Nuomici”, implying an overall acceleration of cell wall breakdown in this cultivar.

### 3.2 IAA and ABA levels in the fruit during preharvest fruit drop

**Figure 2:**
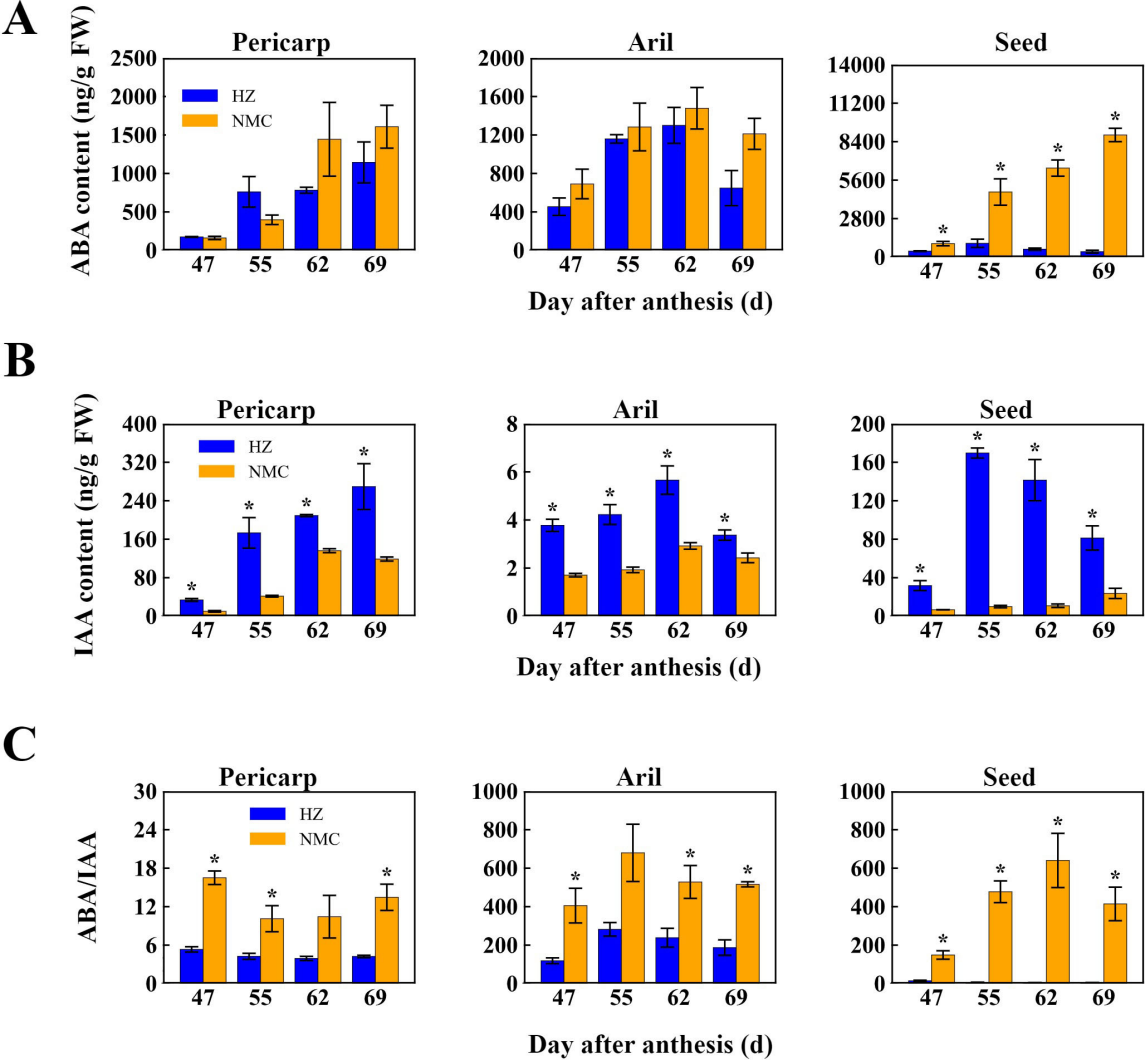
Quantification of abscisic acid (ABA) levels **(A)**, indole-3-acetic acid **(IAA)** levels **(B)**, and the ABA/IAA ratio **(C)** in various fruit tissues of the “Nuomici” (NMC) and “Huaizhi” (HZ) cultivars during preharvest fruit drop. FW denotes fresh weight. Vertical bars represent the standard error of three biological replicates, significant differences at 0.05 level are indicated with one asterisk (*) according to student t-test.

To elucidate the correlation between the preharvest fruit drop rate and the levels of ABA and IAA in different fruit tissues, we measured the contents of these two hormones in the pericarp, aril, and seed and compared their ratios between the “Nuomici” and “Huaizhi” cultivars. In the seed, the ABA levels in “Nuomici” increased gradually during preharvest fruit drop, ranging from 979.82 ng/g FW at 47 DPA to a maximum of 8887.40 ng/g FW at 69 DPA, whereas “Huaizhi” exhibited an initial rise followed by a decrease, reaching a maximum of 1001.77 ng/g FW at 55 DPA (**Figure 2A**). For IAA, in the pericarp, levels in “Nuomici” initially rose and then fell, ranging from 10.07 ng/g FW at 47 DPA to a peak of 136.18 ng/g FW at 62 DPA, while “Huaizhi” steadily increased from 33.79 ng/g FW at 47 DPA to 269.41 ng/g FW at 69 DPA (**Figure 2B**). In the aril, both cultivars exhibited a comparable pattern of IAA levels, with an initial upsurge followed by a decline; “Nuomici” consistently exhibited significantly higher IAA levels than “Huaizhi” during preharvest fruit drop, reaching its peak IAA concentration of 2.92 ng/g FW at 55 DPA, followed by a decline to a minimum of 1.71 ng/g FW at 62 DPA. Regarding the ABA/IAA ratio, “Nuomici” had significantly higher values than “Huaizhi” in all three tissues during preharvest fruit drop (**Figure 2C**). In the pericarp, the ABA/IAA ratio decreased initially and then increased in “Nuomici”, ranging from 10.12 to 16.51, whereas “Huaizhi” remained comparatively stable at 4.6 ± 0.7. In the aril, both cultivars showed an initial increase followed by a decrease, with “Nuomici” and “Huaizhi” reaching maximum ratios of 528.25 and 238.56, respectively, at 55 DPA. In the seed, the ABA/IAA ratio in “Nuomici” exhibited an initial rise followed by a decline, reaching its lowest of 148.81 at 47 DPA and its highest of 640.15 at 62 DPA, whereas “Huaizhi” displayed an inverse pattern, with a minimum of 4.29 at 47 DPA and a maximum of 14.75 at 62 DPA (**Figure 2C**). Overall, these findings suggest that reduced IAA levels in the pericarp, aril, and seed, coupled with elevated ABA levels in the seed, leading to an increased ABA/IAA ratio particularly within the seed, may be positively correlated with preharvest fruit drop in “Nuomici” litchi.

### 3.3 Screening differentially expressed genes associated with preharvest fruit drop in litchi

Significant variations in the dynamics of fruit drop rates, hormone levels, and cellulase activity in the AZ were observed between the “Nuomici” and “Huaizhi” cultivars, indicating distinct molecular regulatory mechanisms. To elucidate these mechanisms, transcriptome sequencing of 96 samples from various fruit tissues and AZ yielded 2421 million high-quality reads, with 86.50% alignment to the litchi genome (refer to **Table S1** and **Supplementary Table 1**). Comparative transcriptomic analyses revealed differentially expressed genes (DEGs) in the pericarp (428), aril (1516), seed (1771), and AZ (1230) between the two cultivars across both inter- and intra-variety (see **Supplementary Figure 1** and **Supplementary Data Sheet 1**). Notably, the number of DEGs increased markedly in each tissue at various timepoints, with the seed showing the most significant changes, rising from 296 to 989, followed by the pericarp (from 53 to 253 DEGs) and the aril (from 434 to 600). In the abscission zone (AZ), the total DEGs continued to increase, from 200 at 55 DPA to 618 at 69 DPA. This substantial upregulation of DEGs across fruit tissues during preharvest fruit drop suggests a comprehensive transcriptional reprogramming underlying the complex physiological cascade leading to fruit drop.

**Figure 3:**
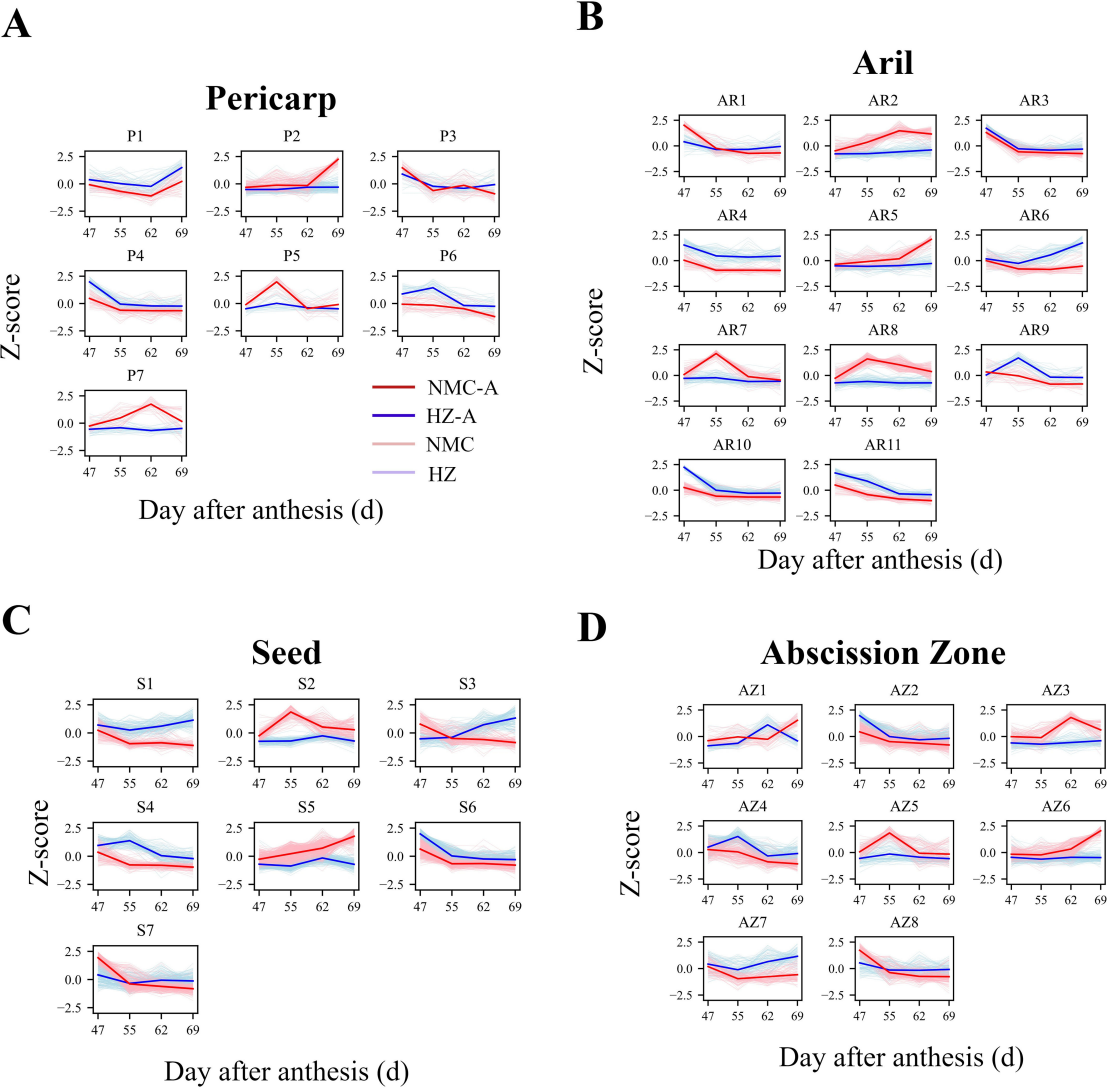
k-means clustering of gene expression patterns in the pericarp **(A)**, aril **(B)**, seed **(C)** and AZ **(D)**. Cluster analysis of DEGs in “Huaizhi” and “Nuomici” based on Z-score normalized method. The light red dotted and light blue dotted represent the expression profiles of “Nuomici” (NMC) and “Huaizhi” (HZ) in each cluster, respectively. The red and blue solid line represent the average profile of “Nuomici” (NMC-A) and “Huaizhi” (HZ-A) in each cluster, respectively.

In light of the differential preharvest fruit drop rates, changes in cellulase activity, and fluctuations in hormone levels within the fruit, we hypothesized that genes positively or negatively correlating with this process would exhibit their peak or lower expression levels, respectively, in “Nuomici” at either 62 or 69 DPA, exhibiting the most pronounced divergence from the expression patterns observed between “Nuomici” and “Huaizhi” during preharvest fruit drop. Therefore, k-means clustering analysis was conducted on DEGs across various tissues to pinpoint those more closely associated with preharvest fruit drop. As shown in **Figures 3A–C**, DEGs in the pericarp, aril, and seed were classified into 7, 11, and 7 clusters, respectively. In the pericarp, Cluster P2 (148 genes) and Cluster P7 (32 genes) corroborated the hypothesis. Cluster P2 exhibited an up-down-up expression pattern in “Nuomici”, contrasting with a plateau-up-plateau trend in “Huaizhi”, with “Nuomici” reaching the highest expression levels at 69 DPA. Cluster P7 displayed an initial uptrend followed by a downtrend in “Nuomici”, whereas it remained relatively stable trend in “Huaizhi”, with the highest expression levels in “Nuomici” at 62 DPA. In the aril, clusters AR2 (119 genes), AR5 (97 genes), and AR6 (66 genes) supported the hypothesis. Cluster AR2 showed an initial increase followed by a decrease in “Nuomici”, whereas it followed a gradual uptrend in “Huaizhi”, with “Nuomici” reaching the highest expression levels at 62 DPA. Cluster AR5 continuously increased in “Nuomici” whereas it showed a down-up-down trend in “Huaizhi”, with “Nuomici” peaking at 69 DPA. Cluster AR6 demonstrated a downtrend followed by an uptrend in “Nuomici”, while in “Huaizhi” it displayed a down-up-down trend, with the lowest expression levels in “Nuomici” at 62 DPA. In the seed, clusters S1 (141 genes), S3 (161 genes), and S5 (353 genes) supported the hypothesis. Cluster S1 exhibited down-plateauing-down trend in “Nuomici”, contrary to an initial decrease followed by an uptrend pattern in “Huaizhi”, with “Nuomici” reaching the lowest expression levels at 69 DPA. Cluster S3 demonstrated a continuous decreasing trend in “Nuomici”, while exhibiting a continuous increasing pattern in “Huaizhi”, with the lowest expression levels in “Nuomici” at 69 DPA. Cluster S5 showed a continuous uptrend in “Nuomici”, but a down-up-down pattern in “Huaizhi”, with the highest expression levels observed in “Nuomici” at 69 DPA. Finally, further refinement based on the k-means clustering analysis identified 180, 282, and 655 DEGs in the pericarp, aril, and seed, respectively.

As illustrated in **Figure 3D**, DEGs in the AZ were classified into 8 clusters, with Clusters AZ3 (88 genes) and AZ6 (153 genes) supporting the hypothesis. Cluster AZ3 exhibited a downtrend followed by an uptrend in “Nuomici”, in contrast to a relatively stable expression profile in “Huaizhi”. The highest expression levels for this cluster were observed in “Nuomici” at 62 DPA. Conversely, Cluster AZ6 demonstrated a continuous uptrend in “Nuomici”, whereas it showed a down-up-down pattern in “Huaizhi”, with the peak expression levels for this cluster occurring in “Nuomici” at 69 DPA. Through this cluster analysis, the study identified a subset of 253 genes in the AZ that merit further investigation to elucidate their potential roles in the underlying mechanisms governing preharvest fruit drop, as their expression patterns significantly diverged between the two cultivars during the critical developmental stages leading up to fruit abscission.

To elucidate the potential roles of differentially expressed genes in preharvest fruit drop, we performed functional classification analysis across different tissues (**Supplementary Figure 2** and **Supplementary Data Sheet 1**). While unclassified genes dominated across all tissues, the abscission zone exhibited significant enrichment in cell wall organization (16) and RNA biosynthesis (16) genes, whereas the seed showed substantial expression in hormone-related genes (27) and RNA biosynthesis (75). The pericarp and aril shared similar patterns in solute transport and enzyme classification, but displayed distinct profiles in cell wall modification genes. These tissue-specific functional distributions, particularly the enrichment of hormone signaling and transcriptional regulation components, provide a theoretical foundation for further investigation of key regulatory networks controlling fruit abscission.

### 3.4 DEGs related to hormone pathways during the preharvest fruit drop

**Figure 4:**
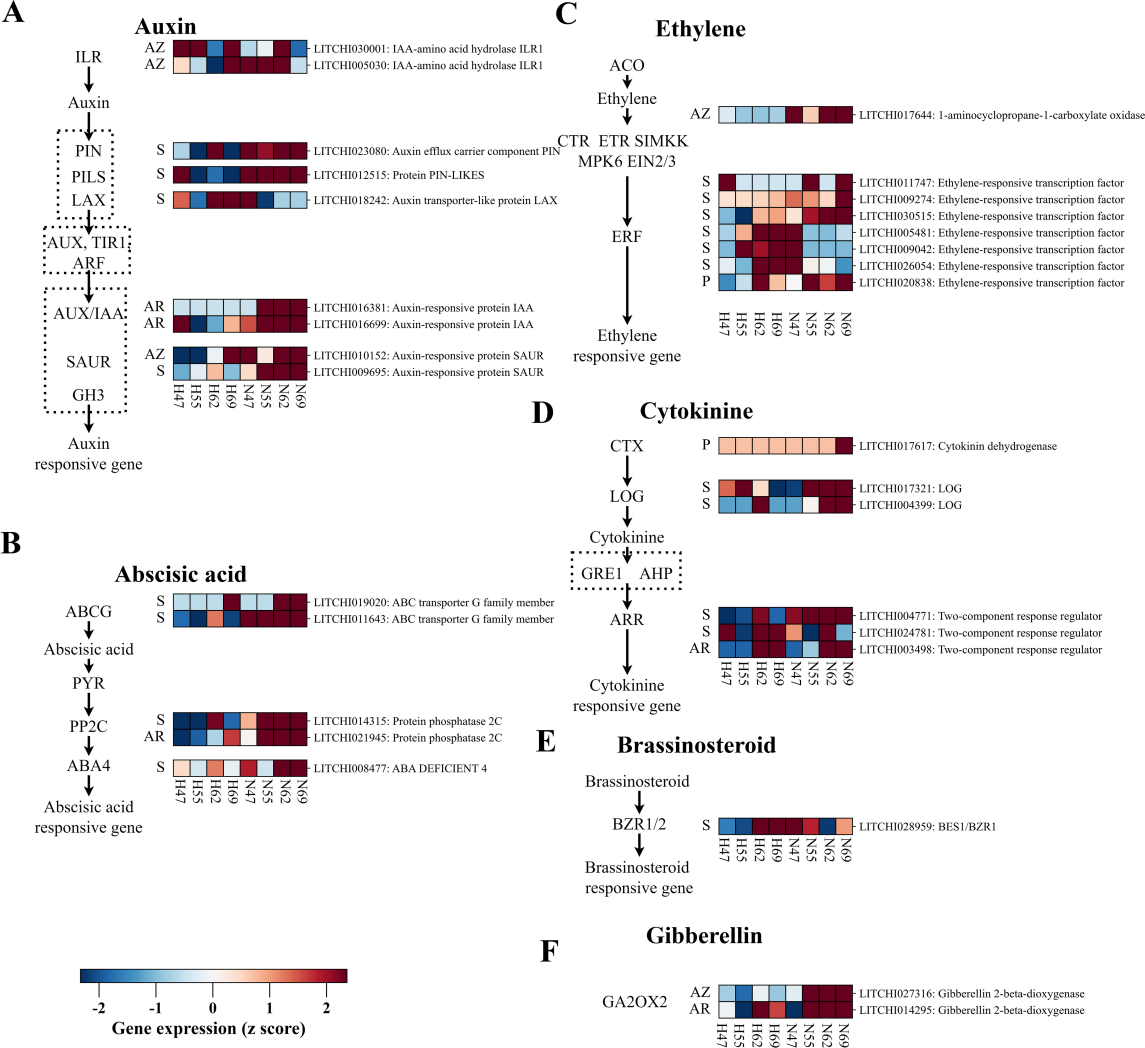
The expression profiling of DEGs related to hormone biosynthesis, transport, and signal transduction pathways between the “Nuomici” and “Huaizhi” cultivars during preharvest fruit drop. The heatmap shows the expression patterns of genes involved in different hormone pathways: auxin **(A)**, abscisic acid **(B)**, ethylene **(C)**, cytokinin **(D)**, brassinosteroid **(E)**, gibberellin **(F)**. The sampling time points and cultivars are indicated at the bottom of the heatmap. “N” and “H” represent the “Nuomici” and “Huaizhi” cultivars, respectively, with the adjacent numbers denoting days post anthesis. The left side of the heatmap displays tissue information as follows: seed (S), pericarp (P), aril (AR), and abscission zone (AZ). The right side of the heatmap displays gene-specific information in the format: gene name: gene annotation. The z-score values are visually represented on a red-blue color scale, where red indicates elevated Fragments Per Kilobase Million (FPKM) values, blue signifies diminished FPKM values, and white corresponds to intermediate FPKM values within the range.

Phytohormones serve as small molecular signal transducers, orchestrate the transmission of abscission cues through a complex interplay of antagonistic and synergistic interactions among various phytohormones. As illustrated in **Figures 4A–E**, 27 DEGs related to hormone biosynthesis, transport, and signal transduction across various fruit tissues were identified. In the pericarp, two DEGs were upregulated in “Nuomici”: 1) a cytokinin oxidase/dehydrogenase gene (*LITCHI017617*) associated with CTK homeostasis, and 2) an ethylene response factor (*LITCHI020838*) related to ethylene signaling. In the aril, six DEGs were higher expressed in “Nuomici”, distributed across different hormone pathways: 1) a gene encode protein phosphatase 2C (*LITCHI021945*) related to ABA response, 2) a gene encoding gibberellin 2-beta-dioxygenase (*LITCHI014295*) related to gibberellic acid homeostasis, 3) a gene encoding two-component response regulator (*LITCHI003498*) related to cytokinin response, and 4) two DEGs encoding auxin-responsive protein IAA (*LITCHI016699* and *LITCHI016381*) related to associated with IAA response. The seed demonstrated the most complex regulation, with 23 DEGs identified in phytohormone pathways. Of these, 16 DEGs and 7 DEGs exhibited higher and lower expression in “Nuomici”, respectively. Notably, auxin-related genes showed varied expression patterns: 1 DEG (auxin-responsive protein SAUR: *LITCHI009695*) involved in IAA response was higher expressed in “Nuomici”, while 3 DEGs related to IAA transport exhibited mixed patterns. Among these three DEGs related to IAA transport, the PIN-LIKES gene (*LITCHI012515*) and the auxin efflux carrier component PIN (*LITCHI023080*) were upregulated in the seed of “Nuomici”, whereas the gene encoding auxin transporter-like protein LAX (*LITCHI018242*) was downregulated in the seed of “Nuomici”. Ethylene response genes in the seed showed intricate regulation, with three ethylene-responsive transcription factors (*LITCHI030515*, *LITCHI009274*, *LITCHI011747*) exhibiting higher and three ethylene-responsive transcription factors (*LITCHI005481*, *LITCHI026054*, *LITCHI009042*) showing lower expression in “Nuomici”. Additionally, two DEGs (ABA DEFICIENT 4: *LITCHI008477*, protein phosphatase 2C: *LITCHI014315*) related to ABA response and two DEGs (ABC transporter G family member: *LITCHI019020* and *LITCHI011643*) related to ABA transport exhibited higher expression in “Nuomici”. Cytokinin-related genes also displayed diverse expression patterns: two DEGs (two-component response regulator: *LITCHI024781* and *LITCHI004771*) related to cytokinin response showed mixed expression, while two DEGs (LOG: *LITCHI004399* and *LITCHI017321*) involved in cytokinin balance exhibited higher expression in the seed of “Nuomici”. Furthermore, one DEG (BES1/BZR1: *LITCHI028959*) related to brassinosteroids response was observed to be lower expressed in the seed of “Nuomici”, while one DEG (gibberellin 2-beta-dioxygenase: *LITCHI028959*) related to gibberellin balance showed higher expression in the seed of “Nuomici”. In conclusion, this complex interplay of phytohormone-related gene expression across different fruit tissues highlights the intricate and interconnected mechanisms of abscission signal generation and transduction governing the abscission process.

Upon detection of the abscission cues from the fruit, the AZ subsequently coordinates the conversion of these signals into the activation of the abscission process through a delicate interplay of various phytohormones. As shown in **Figure 4**, five DEGs associated with phytohormone pathways were identified in the AZ. The majority of these DEGs exhibited reduced expression in the AZ of “Nuomici”. Notably, one DEG (auxin-responsive protein SAUR: *LITCHI010152*) related to IAA response and two DEGs (IAA-amino acid hydrolase ILR1: *LITCHI005030* and *LITCHI030001*) related to IAA balance exhibited increased expression in “Nuomici”. Additionally, one DEG (1-aminocyclopropane-1-carboxylate oxidase: *LITCHI017644*) related to ethylene synthesis and one DEG (gibberellin 2-beta-dioxygenase: *LITCHI027316*) related to gibberellin balance exhibited higher expression in the AZ of “Nuomici”. This complex interconnection of phytohormone-related gene expression in the AZ suggests that upon receiving fruit abscission signals, the AZ undergoes a disruption in hormonal equilibrium, indicating sophisticated crosstalk among multiple phytohormones signaling pathways in regulating this vital physiological event.

### 3.5 Transcription factors in abscission zone associated with preharvest fruit drop

**Figure 5:**
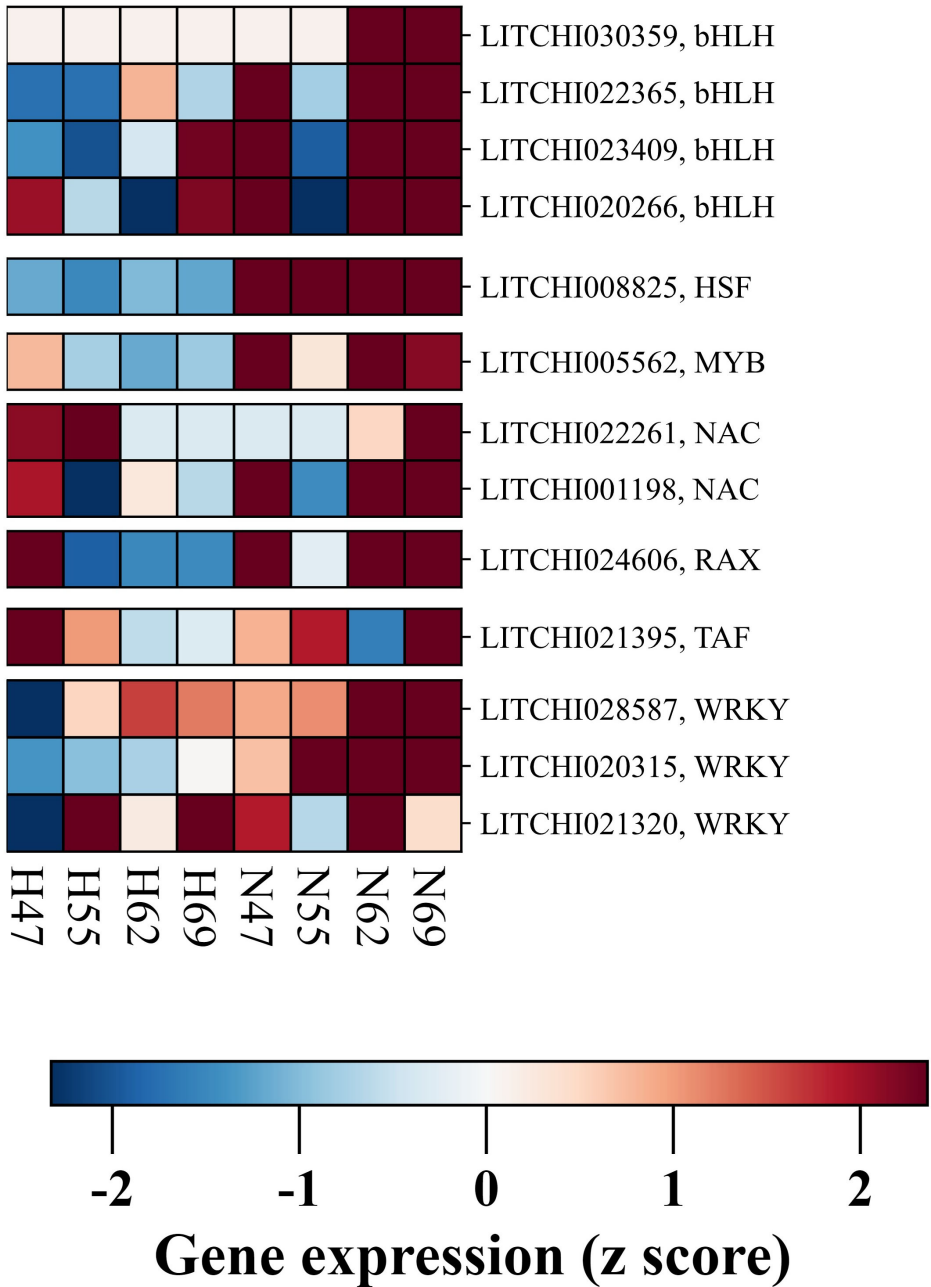
Expression profiling of DEGs related to transcriptional factors in the abscission zone (AZ) between “Nuomici” and “Huaizhi” during preharvest fruit drop. “N” and “H” represent the “Nuomici” and “Huaizhi” cultivars, respectively, with the adjacent numbers denoting days post anthesis.

Transcription factors (TFs) are pivotal in orchestrating the complex process of preharvest fruit abscission. Our study has identified eighteen DEGs associated with transcriptional regulation, unveiling a complex regulatory landscape (**Figure 5**). Three prominent TF families emerged with multiple DEGs: the WRKY family (three DEGs: LITCHI020315, LITCHI021320, LITCHI028587), the NAC family (two DEGs: LITCHI001198, LITCHI022261), and the bHLH family (four DEGs: LITCHI020266, LITCHI022365, LITCHI023409, LITCHI030359). All WRKY genes exhibited elevated expression in the AZ of “Nuomici”, reaching a peak at 62 DPA, with one gene (LITCHI021320) showing a subsequent decline at 69 DPA. Among the NAC genes, one (LITCHI001198) maintained high expression from 62 DPA onwards in the AZ of “Nuomici”, while the other (LITCHI022261) peaked at 69 DPA. All bHLH genes demonstrated upregulation at both 62 and 69 DPA. Additionally, single DEGs from several other TF families were identified, including HSF (LITCHI008825), MYB (LITCHI005562), RAX (LITCHI024606) and TAF (LITCHI021395). For these single-gene TF families, HSF, MYB and RAX all peaked in the AZ of “Nuomici” at 62 DPA. TAF, however, exhibited its highest expression at 69 DPA. Notably, the majority of TFs associated with preharvest fruit abscission showed elevated expression by 62 DPA, indicating a critical regulatory period preceding the onset of abscission. The predominance of transcriptional regulatory processes prior to the peak of preharvest fruit drop suggests a proactive genetic orchestration of abscission. This potentially initiates a cascade of molecular and physiological events culminating in fruit detachment, underscoring the critical importance of early intervention strategies in mitigating yield losses.

### 3.6 DEGs related to reactive oxygen species, programmed cell death, and metabolisms of cell wall modification during preharvest fruit drop

**Figure 6:**
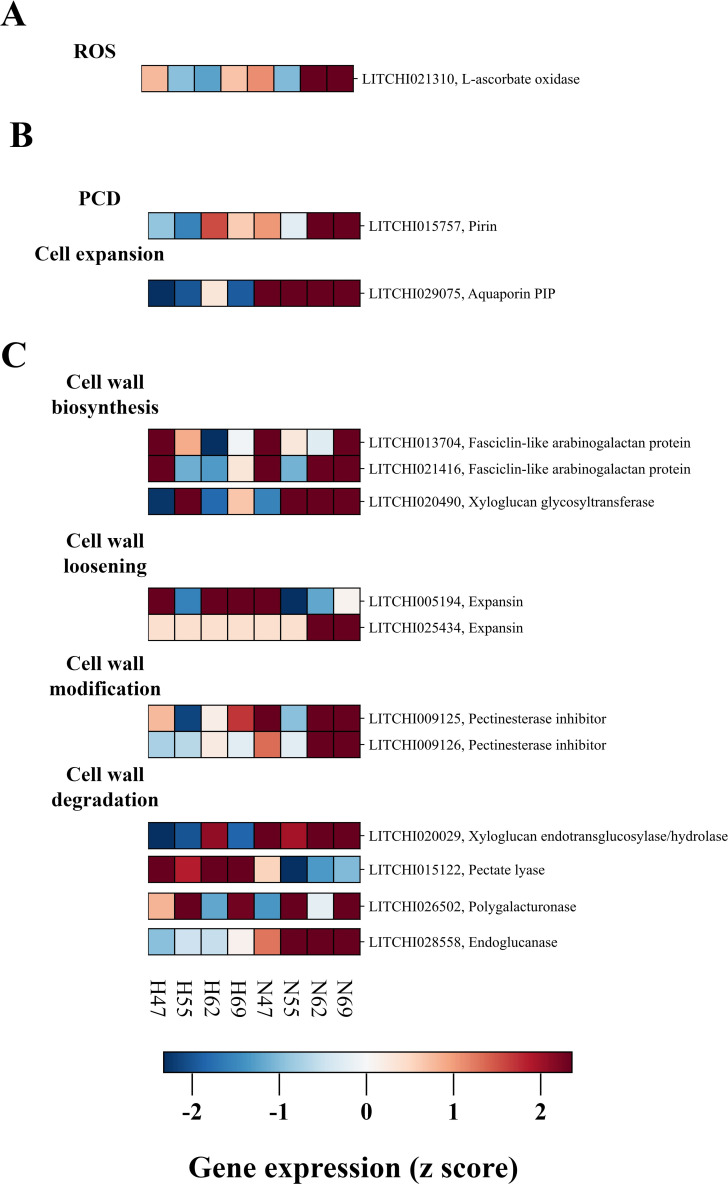
Expression profiling of DEGs related to ROS **(A)**, PCD **(B)**, cell wall metabolism **(C)** in abscission zone between “Nuomici” and “Huaizhi” during preharvest fruit drop. “N” and “H” represent the “Nuomici” and “Huaizhi” cultivars, respectively, with the adjacent numbers denoting days post anthesis.

The process of abscission is often accompanied by a burst of reactive oxygen species (ROS) within the fruit AZ (Yang et al., 2015). As illustrated in **Figure 6A**, one DEG encoding L-aldehyde oxidase (LITCHI021310), which is implicated in ROS production, exhibited higher expression in the AZ of “Nuomici”. This expression profile suggests a potential imbalance in the ROS production and scavenging within the AZ, leading to ROS accumulation and an oxidative burst, which may serve as a crucial signaling event triggering cell separation in litchi.

The dissolution of the AZ is inherently associated with the induction of programmed cell death (PCD) and a reorganization of the cell wall architecture within this specialized tissue. This study identified one Prin gene (*LITCHI015757*) associated with PCD and one aquaporin PIP gene (*LITCHI029075*) related to cell expansion that exhibited higher expression in the AZ of “Nuomici” (**Figure 6B**). In addition to the DEGs closely associated with PCD and cell expansion, we observed differential expression patterns in sixteen DEGs involved in cell wall organization, as shown in **Figure 6C**. Notably, these DEGs were involved in a variety of cellular processes related to cell wall modification, loosening, degradation, and biosynthesis. Regarding cell wall loosening, two DEGs (*LITCHI022703* and *LITCHI027667*) encoding expansin proteins exhibited higher expression in the AZ of “Nuomici”. Multiple DEGs were implicated in cell wall biosynthesis and modification, including one xyloglucan glycosyltransferase (*LITCHI020490*), two fasciclin-like arabinogalactan (*LITCHI021416* and *LITCHI013704*), and two pectinesterase inhibitor genes (*LITCHI009126*, *LITCHI009125*). Furthermore, four DEGs (endoglucanase: *LITCHI028558*, polygalacturonase: *LITCHI026502*, pectate lyase: *LITCHI027260*, xyloglucan endotransglucosylase/hydrolase: *LITCHI020029*) were involved in cell wall degradation processes, indicating active remodeling of cell wall components. These findings collectively highlight the intricate transcriptional regulation of various cell wall-associated processes in the AZ during the preharvest drop stage. The identification of DEGs involved in cell expansion, cell wall loosening and degradation, along with those associated with PCD and cell expansion, suggests that the AZ is undergoing concurrent remodeling of the cell wall architecture and execution of programmed cell death cascades, potentially facilitating the abscission process.

### 3.7 Enrichment analysis and co-expression network analysis of differentially expressed genes in preharvest fruit drop

**Figure 7:**
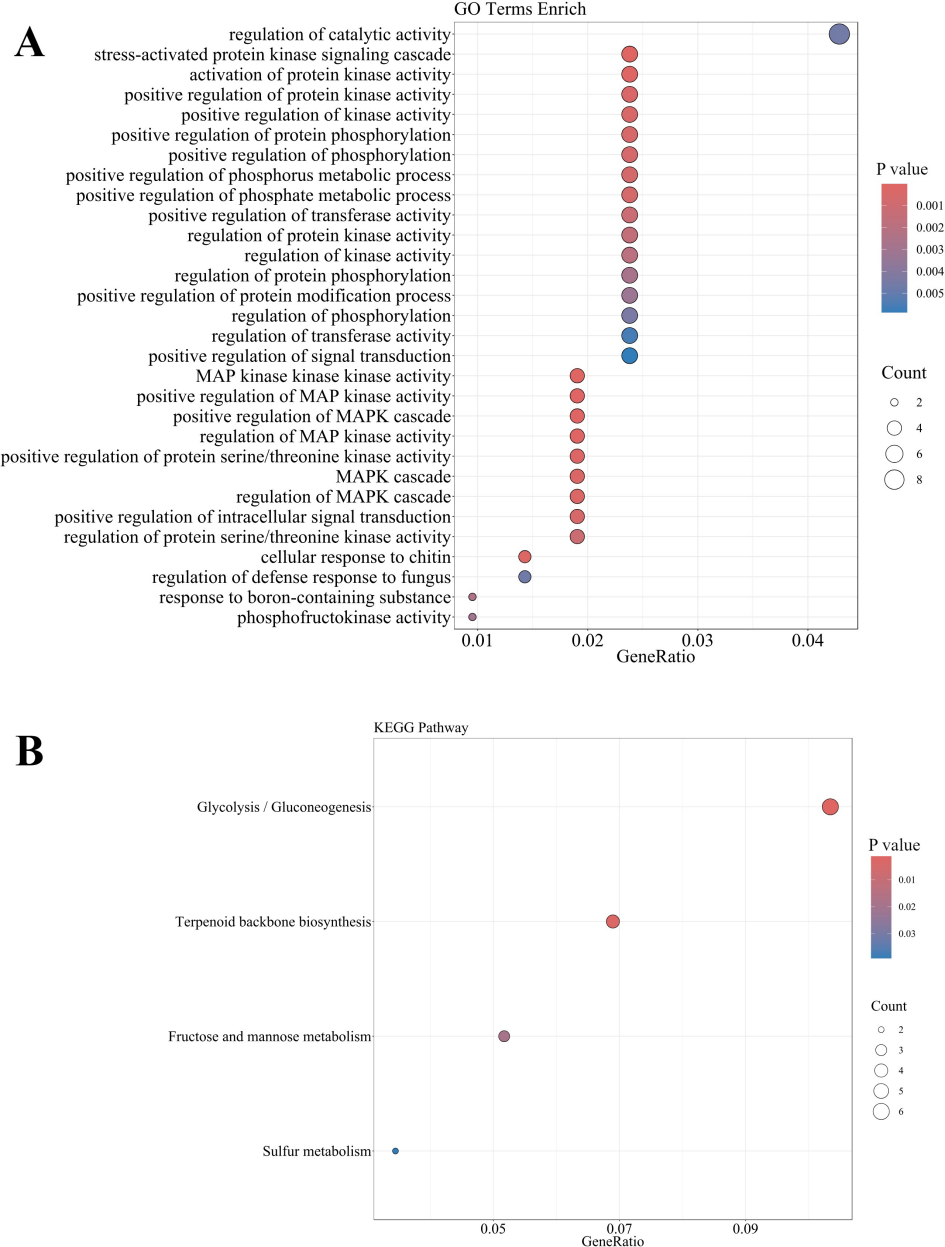
GO **(A)** and KEGG **(B)** enrichment analysis of DEGs in the abscission zone during preharvest fruit drop.

To elucidate the molecular mechanisms underlying preharvest fruit abscission, we performed GO enrichment analysis, KEGG pathway mapping, and co-expression network analysis of differentially expressed genes in the abscission zone. GO enrichment analysis (**Figure 7A**) revealed significant enrichment in hormone signal transduction (GO:0000165), stress response pathways (GO:0031669, GO:0001666), and cellular redox homeostasis (GO:0045454), along with multiple terms related to signal transduction, including regulation of kinase activity (GO:0043549) and protein phosphorylation (GO:0001932). Complementary KEGG pathway analysis (**Figure 7B**) further substantiated these findings by revealing the involvement of glycolysis/gluconeogenesis (ko00010), fructose and mannose metabolism (ko00051), terpenoid backbone biosynthesis (ko00900) and sulfur metabolism (ko00920) processes during fruit abscission. These functional enrichment analyses highlight the intricate interplay between hormone-mediated signaling cascades and metabolic reprogramming during the abscission process.

Hierarchical analysis of Gene Ontology terms (**Supplementary Figure 3** and **Supplementary Data Sheet 1**) revealed a significant enrichment cascade centered on MAPK signaling, with the most significant terms including callose-mediated defense response regulation (GO:2000071, *p* = 2.6e-05) and MAPK activity regulation (GO:0043406, *p* = 6.5e-05). This hierarchical organization suggests that MAPK-mediated phosphorylation cascades serve as a central regulatory hub integrating stress responses with protein kinase signaling networks, potentially triggering downstream cellular responses that ultimately lead to preharvest fruit abscission. The prominence of stress-activated protein kinase signaling (GO:0031098) further indicates that preharvest fruit drop may be initiated through stress-induced signal transduction cascades. These hierarchical relationships reveal a sophisticated stress response mechanism where MAPK signaling orchestrates multiple cellular processes during fruit abscission.

Co-expression network analysis uncovered two distinct regulatory modules (**Supplementary Figure 4** and **Supplementary Data Sheet 1**): the first module demonstrated strong co-expression relationships between bHLH (*LITCHI022365*), WRKY (*LITCHI021320*), PRIN (*LITCHI015757*), and TAF (*LITCHI021395*) transcription factors with ILR (*LITCHI030001*) and ACO (*LITCHI017644*) genes involved in hormone signaling and programmed cell death, while the second module revealed coordinated expression between NAC (*LITCHI001198*), MYB (*LITCHI005562*), and HSF (*LITCHI008825*) transcription factors and cell wall metabolism-related genes, particularly XTH (*LITCHI020029*) and PMEI (*LITCHI091125*). These co-expression patterns indicate a hierarchical transcriptional regulation system where specific transcription factor families coordinate distinct aspects of the abscission process. The modular organization of these networks suggests a precise temporal and spatial control of abscission-related processes through coordinated transcriptional regulation.

Collectively, our comprehensive analyses reveal a multi-layered regulatory network underlying preharvest fruit abscission, where stress-activated MAPK cascades integrate environmental signals with hormone responses, leading to metabolic reprogramming and cell wall modifications through the coordinated action of specific transcription factor modules. This intricate regulatory system appears to fine-tune the abscission process through the orchestration of multiple cellular pathways, providing potential targets for manipulating fruit retention in agricultural practice.

### 3.8 Validation of RNA-seq data

**Figure 8:**
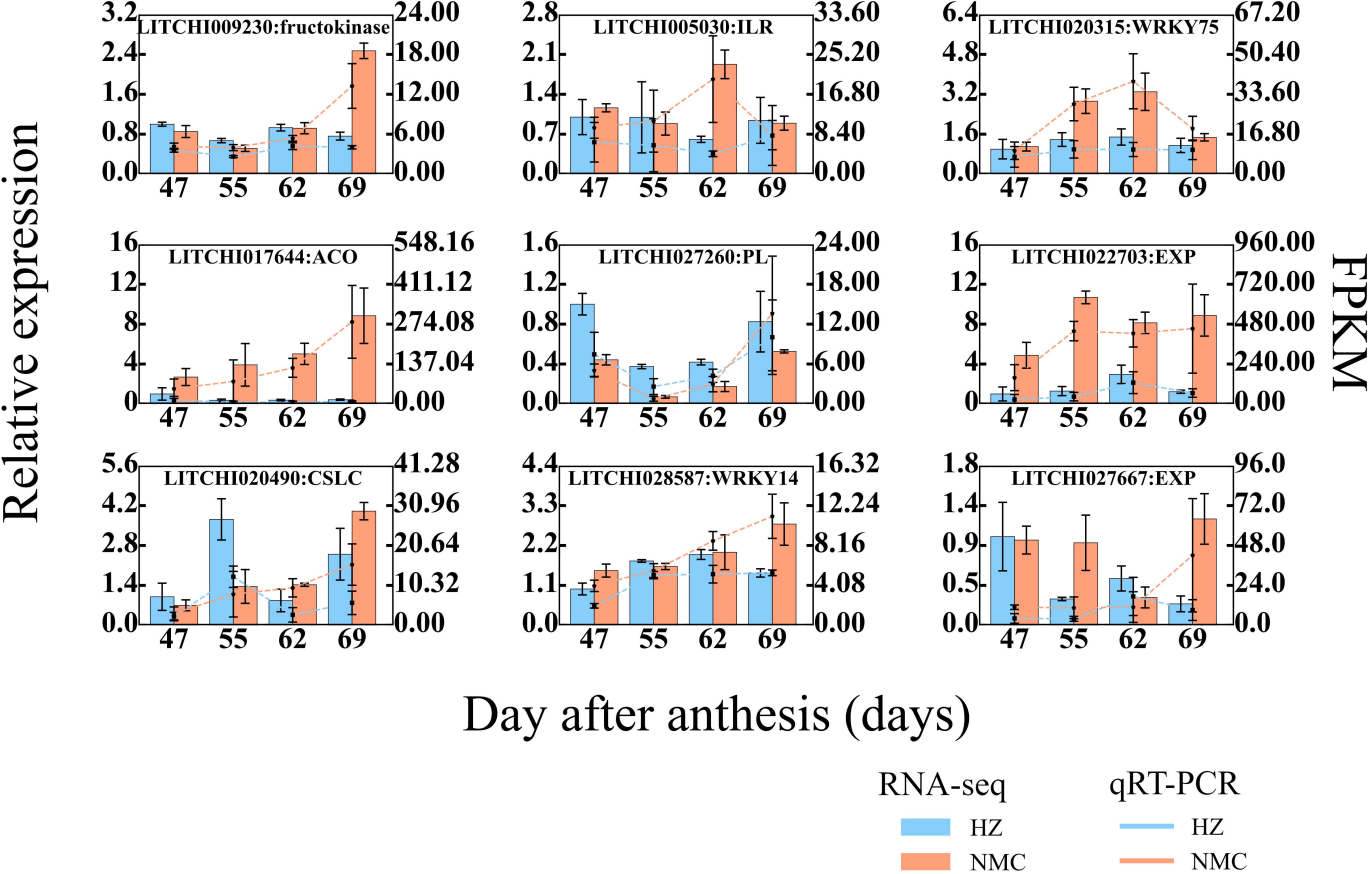
qRT-PCR (Quantitative Real-time Polymerase Chain Reaction) analysis of 9 randomly selected genes in abscission zone. Lc-Actin-1α were used as reference genes for normalization of gene-expression data. Red and blue bars represent the data yielded by qRT-PCR in “Nuomici” and “Huaizhi”, respectively. Red and blue lines represent the data yielded by RNA sequencing in “Nuomici” and “Huaizhi”, respectively. Error bars indicate standard errors of the means (n = 3).

To verify the reliability of the RNA-seq data, 9 DEGs in AZ were randomly selected for qRT-PCR validation. As shown in **Figure 8** (**Table S2** and **Supplementary Table 1**), the qRT-PCR results confirmed that the expression patterns of all DEGs in AZ were consistent with the RNA-seq findings. These DEGs exhibited higher expression levels in the AZ of “Nuomici” included ILR (*LITCHI005030*), WRKY75 (*LITCHI020315*), fructokinase (*LITCHI009230*), ACO (*LITCHI 017644*), EXP (*LITCHI 027667*, *LITCHI022703*), and WRKY14 (*LITCHI028587*), while CSLC (*LITCHI020490*) and PEL (*LITCHI027260*) were exhibited lower expression levels in “Nuomici”. Collectively, these findings demonstrate the reliability and consistency of the transcriptomic data, providing a robust foundation for further investigations into the molecular mechanisms underlying preharvest fruit drop in litchi.

## 4 Discussion

Certain well-known litchi varieties, such as “Nuomici”, frequently suffer severe preharvest fruit drop, which results in considerable yield losses. Research has indicated that “Nuomici” is particularly susceptible to fruit drop prior to harvest, with losses ranging from 20%-50% (Zhao and Li, 2020). Nevertheless, the molecular basis of preharvest fruit drop in litchi remains poorly understood, particularly the mechanism by which signals prompting abscission are transmitted from the fruit to the abscission zone (AZ) and initiate fruit detachment. In this study, we employed simultaneous RNA sequencing to analyze fruit and AZ tissues, complemented by phytohormone quantification and enzyme activity assessments. This approach enabled us to propose a molecular model in which the fruit exerts a “remote control” over the AZ during the onset of litchi preharvest fruit drop.

### 4.1 Hormonal balance of IAA and ABA in fruit as potential abscission cues

When fruit trees are exposed to adverse biotic or abiotic signals, they can generate abscission cues that are then transmitted to the pedicel abscission zone (AZ), leading to fruit drop. Among the various signals, plant hormones, particularly IAA and ABA, play a vital role in the generation and transmission of abscission cues during fruit abscission (Botton et al., 2011; Else, 2004; Zhao and Li, 2020). IAA in the fruit primarily regulates fruit abscission by directly influencing polar auxin transport in the AZ and altering its sensitivity to abscission cues such as ethylene (Zhao et al., 2024). The abscission rate of citrus pedicel increased sharply to 77% at eight days after the ovary was removed, while the calyxes with retained fruitlets or IAA treatment did seldom abscise (Xie et al., 2018). Directly spraying 15mg/L of 2,4-dichlorophenoxyacetic acid (2,4-D) before mature fruit abscission can increase the content of auxin and its analogues, thereby reducing preharvest fruit drop in sweet oranges (*Citrus sinensis* L. Osbeck) “*Washington navel*” and “*Navelate*” (Agustí et al., 2009). In litchi, 2,4-D treatment could also prevent the fruitlet abscission (Peng et al., 2013). In this study, the IAA content in the whole fruit of “Nuomici” was significantly lower than that of “Huaizhi” during preharvest fruit drop, indicating that the IAA content in the fruit may disrupt the polar auxin transport (PAT) within the AZ, making it more sensitive to abscission cues such as ethylene. ABA in the fruit can activate other abscission cues and transmit them to the AZ, inducing fruit drop. In apple, ABA levels strongly correlate with isoprene production in the apple fruit cortex, which serves as an early indicator of abscission induction (Giulia et al., 2013). Spraying ABA can induce fruitlet abscission in peach, but this process is accompanied by an increase in ethylene synthesis (Giovanaz et al., 2015). In litchi, the occurrence of preharvest fruit drop was accompanied by an increase in ABA level in the seed and aril (Xiang et al., 1995; Yuan and Huang, 1988). In the current study, the ABA content in the seed of “Nuomici” was in accordance with the dynamics of preharvest fruit drop and significantly different from that of “Huaizhi”, suggesting that ABA in the seed may be one of factors inducing preharvest fruit drop. In addition to the roles of individual hormones, the balance between ABA and growth-promoting hormones is closely associated with preharvest fruit drop. Qiu et al. (1998) compared three litchi cultivars with different patterns of preharvest fruit drop and discovered that a higher relative ratio of (IAA+GAs+CTK)/ABA was closely correlated with reduced preharvest fruit drop. This suggests that the intricate interplay between ABA and growth-promoting hormones, rather than their absolute levels, may be a critical determinant of preharvest fruit retention in litchi. Notably, this study found that the most pronounced difference in the hormonal ratio was observed in the seed (**Figure 2C**), suggesting that the hormonal imbalance, particularly the elevated ABA/IAA ratio in the seed (**Figures 2A, B**), may be most closely associated with the enhanced preharvest fruit drop in “Nuomici”. Discrepancies in the ABA/IAA ratio were also evident, though to a lesser extent, in the pericarp and aril tissues. Overall, these hormonal discrepancies likely act in combination as potential abscission cues. The lower auxin levels across the fruit enhance the sensitivity of the abscission zone (AZ) to these cues, while the higher ABA content in the seed activates other abscission signaling pathways. This collective hormonal dysregulation facilitates the enhanced preharvest fruit drop observed in “Nuomici”.

### 4.2 The interplay among the hormones is involved in the transmission of the abscission signal from the fruit to the abscission zone

In plants, the transport of indole-3-acetic acid (IAA) predominantly occurs through two mechanisms: active transport, which is mediated by AUX1/LAX, PIN, and ABCB proteins, requires energy and exhibits directional intercellular movement, and passive transport, including concentration diffusion through various pathways, which is non-directional and does not require energy (Vanneste and Friml, 2009). A comparative study of transcriptomic changes in seeds from different fruitlet types revealed that the expression dynamics of IAA transport protein *MdPIN1* indicates greater auxin transport in central fruit seeds (Ferrero et al., 2015). In this study, we identified two auxin transport proteins, PIN (*LITCHI023080*) and PILS (*LITCHI012515*), that were upregulated in the seeds of “Nuomici”, while one LAX gene (*LITCHI018242*) was downregulated, indicating a higher auxin transport rate in the seed of “Nuomici” during the preharvest fruit drop (**Figure 4A**). This is in contrast to the findings of Ferrero et al. (2015) in apples, suggesting that “Nuomici” seeds may undergo more frequent developmental terminations, which could require lower auxin levels and thus result in a higher export of IAA from the seed. IAA-amino acid hydrolase cleaves the conjugated bonds of IAA conjugates, thereby increasing free IAA content (LeClere et al., 2002). We found that two ILR genes (*LITCHI030001*, *LITCHI005030*) exhibited lower expression levels in the AZ of “Nuomici” at 69 DPA. This reduction in total IAA levels suggests a potential link between diminished IAA homeostasis and preharvest fruit drop in the AZ. ARFs and AUX/IAA proteins heterodimerize to regulate auxin-responsive gene expression, with SAURs and GH3 genes acting as downstream ARF targets, collectively modulating auxin signaling and plant developmental responses (Li et al., 2023; Tahir et al., 2023). However, evidence from transgenic overexpression studies indicates that rice SAUR39 functions as a negative regulator of auxin biosynthesis and transport, leading to reduced auxin levels and altered plant growth consistent with auxin deficiency (Kant et al., 2009). In our study, the SAUR genes (*LITCHI010152*, *LITCHI009695*) identified in both seed and AZ tissues, are more highly expressed in “Nuomici” and likely to function as negative regulators of auxin biosynthesis and transport. This is consistent with the observed lower auxin content in the seeds, suggesting a crucial role in modulating auxin homeostasis during preharvest fruit drop. Notably, the absence of one ARF genes (*LITCHI016495*), previously identified by Ma et al. (2024), suggests potential variations in the regulatory mechanisms governing fruitlet abscission and preharvest fruit drop, highlighting the complexity of abscission control across different developmental stages. In brief, fluctuations in seed auxin levels may modulate the efficiency of auxin transport and trigger signaling cascades in the aril that propagate abscission signals to the AZ, where altered hormone homeostasis initiates the abscission process.

The influence of ABA on fruit abscission is well-documented, but the exact mechanisms of its signaling pathways have not yet been fully elucidated. Application of exogenous ABA to citrus branches results in a transient increase in IAA levels in the AZ, which promotes ethylene production and thereby accelerates fruit abscission (Okuda, 1999). A comparable phenomenon was observed in the leaf AZ during olive leaf abscission (Kitsaki et al., 1999). ABC transporters, particularly those of the ABCG subfamily, are essential for ABA transport (Do et al., 2018). In this study, the upregulation of two ABCG transporter genes (*LITCHI011643*, *LITCHI019020*) in the seed of “Nuomici” suggests enhanced ABA transport from the seed to other tissues, such as the aril and the AZ, potentially mediating ABA-dependent processes during preharvest fruit drop. Once ABA reaches its target locations, it mediates downstream signaling responses through the well-characterized PYR/PYL/RCAR-|PP2C-|SnRK2 pathway (Shi et al., 2022). In this study, we identified two and one DEGs associated with ABA signaling in the seed and aril, respectively. In the seed of “Nuomici”, one PP2C (*LITCHI014315*) and one ABA4 (*LITCHI008477*) exhibited higher expression (**Figure 4B**), indicating that the higher ABA levels in the seed of “Nuomici” may trigger the activation of specific ABA signaling pathways. The higher expression of PP2C (*LITCHI021945*) in the aril of “Nuomici” suggests that the higher ABA levels in the seed may be partially transported to the aril, inducing the activation of the ABA signaling pathway. In summary, the identification of ABCG proteins associated with ABA transport in the seed of “Nuomici”, coupled with the higher ABA levels and higher expression of ABA signaling genes in both seed and aril, suggests a potential transport of ABA from the seeds to the aril, where it may participate in the abscission process.

Ethylene (ET) is a key hormone regulating plant organ abscission, and the balance between ET and IAA dynamics is critical for determining whether AZ cells separate or not. When the polar transport of IAA in the AZ is disrupted or IAA decreases, it can stimulate ET production in the AZ and increase its sensitivity to ET. Our previous studies have confirmed that the release of ethylene from the fruit is a key factor in litchi fruitlet abscission (Li et al., 2015b). In this study, one ACO gene (*LITCHI017644*) was found to be highly expressed in the AZ of “Nuomici” (**Figure 4C**), indicating that the AZ may produce more ethylene during the preharvest fruit drop stage. Interestingly, the ACO gene identified in this study is different from *LcACO2/3* (*LITCHI029670*, *LITCHI011290*) which are involved in fruitlet abscission (Ma et al., 2019), suggesting that there may be differences in ethylene synthesis between preharvest fruit drop and fruitlet abscission. EIN, ETR and ERF are essential ethylene response genes in the ethylene signal transduction process (Liu et al., 2015). In this study, one ERF gene (*LITCHI020838*) was found to have higher expression, suggesting that the ethylene signaling pathway in the pericarp of “Nuomici” may be indirectly modulated by other hormones acting on the ERF transcription factor. However, the differential expression patterns of the six identified ERF genes in the seed, with three DEGs (*LITCHI011747*, *LITCHI009274*, *LITCHI030515*) upregulated and three DEGs (*LITCHI005481*, *LITCHI009042*, *LITCHI026054*) downregulated in “Nuomici”, suggest a complex regulatory network governing ethylene response, potentially modulating the interplay between ethylene and other hormones during fruit development. Furthermore, other ethylene response genes involved in litchi fruitlet abscission, such as *LcEIL2/3* (Ma et al., 2020), *LcERF2* (*LITCHI027926*) (Yi et al., 2021), *LcERF10* (*LITCHI010942*) (He et al., 2023), were not identified in this study, indicating significant differences between preharvest fruit drop and fruitlet abscission in litchi. The lack of previously identified fruitlet abscission-related ERF genes in the AZ during preharvest fruit drop suggests a more complex, multi-ERF regulatory mechanism in mature fruit abscission, in contrast to the dominant role of single ERF genes observed in fruitlet abscission in litchi. These findings collectively highlight the intricate and developmentally specific nature of ethylene-mediated abscission in litchi, revealing distinct regulatory mechanisms between preharvest fruit drop and fruitlet abscission, and emphasizing the need for targeted, developmentally specific strategies for managing fruit retention.

In addition to auxin, ABA, and ethylene, our study also identified genes related to cytokinin (CTK), brassinosteroids and gibberellin that may be involved in preharvest fruit drop. Through four separate experiments, applying 200 mg/L before anthesis significantly increased initial fruit set rates at 2 weeks after anthesis, from 8-17% in control racemes to 27-61% in the treated racemes (Trueman, 2010). The homeostasis of endogenous cytokinin is tightly regulated by various enzymes, including cytokinin oxidase/dehydrogenase, which catalyzes the irreversible degradation of CTK (Schmülling et al., 2003). On the other hand, LONELY GUY (LOG) genes play a crucial role in CTK activation. Tsaniklidis et al. (2016) demonstrated that conditional overexpression of *AtLOGs* in transgenic *Arabidopsis* plants reduced N6-(Δ2-isopentenyl)adenine (iP) riboside 5’-phosphates while increasing the levels of iP and its glucoside derivatives. Our study identified one cytokinin oxidase/dehydrogenase gene (*LITCHI017617*) exhibited higher expression in the pericarp of “Nuomici” during preharvest fruit drop (**Figure 4D**), suggesting accelerated irreversible degradation of endogenous CTK compared to “Huaizhi”. Additionally, two LOG genes (*LITCHI017321* and *LITCHI004399*) were found to have higher expression levels in the seed of “Nuomici” during preharvest fruit drop, indicating that most of the remaining CTK in the seed of “Nuomici” was in an activated state. Arabidopsis response regulators (ARRs) have species-specific functions in CTK signal transduction, including positive regulation of axillary bud outgrowth, nuclear localization, and potential interaction with MADS-box transcription factors (Zhao et al., 2023). Notably, this study identified one and 2 ARR genes expressed differentially in the aril and another two in the seed. One ARR gene (*LITCHI004771*) was found to have lower expression in the aril, indicating suppressed CTK signaling transduction in these tissues. The differing expression patterns of two ARR genes (*LITCHI003498*, *LITCHI024781*) in the seed suggested a complex CTK signaling mechanism, necessitating further investigation to elucidate the intricate regulatory network governing seed development during preharvest fruit drop. Brassinosteroids have been shown to inhibit ethylene biosynthesis and reduce fruit abscission by positively regulating BZR1/2 in the AZ (Ma et al., 2021). Our study identified One BZR1 gene (*LITCHI028959*) was found to have lower expression in the seed of “Nuomici” (**Figure 4E**), implying its role in ethylene regulation within the fruit. 2-oxoglutarate-dependent dioxygenases known as GA 2-oxidases (GA2oxs) are key to the inactivation of bioactive gibberellins (Li et al., 2019). In this study, one GA2ox gene (*LITCHI028959*) was identified as being more highly expressed in the seeds of “Nuomici” (**Figure 4F**), suggesting enhanced inactivation of bioactive gibberellins in the seed tissue. Concurrently, another GA2ox gene (*LITCHI027316*) exhibited higher expression in the AZ of “Nuomici”, indicating a similar reduction in bioactive GA levels in this region. The simultaneous decrease in bioactive GA across both seed and AZ may serve as a critical signaling mechanism initiating the abscission process. This coordinated reduction in bioactive GA levels across these distinct tissues may represent a key regulatory component in the complex cascade leading to fruit drop. Overall, these findings highlight the tissue-specific dynamics of CTK homeostasis and signaling, possibly regulated by crosstalk with other phytohormonal pathways during seed development and abscission processes.

### 4.3 Key transcriptional factors associated with preharvest fruit drop in abscission zone

The phenomenon of preharvest fruit drop is characterized by extensive gene transcription, which requires regulation by a diverse array of transcription factors. Extensive research has demonstrated that transcriptional regulation by these factors is an essential part of the abscission process, such as in tomato flower and fruit abscission (Gao et al., 2016; Meir et al., 2010; Nakano et al., 2012), citrus fruitlet abscission (Xie et al., 2018), melon mature fruit abscission (Corbacho et al., 2013), and sweet orange fruit abscission (Zhao et al., 2019). In litchi, studies have identified differential expression of transcription factors such as bHLH, NAC, MYB, WRKY, and LBD during fruitlet abscission (Li et al., 2015a, b). Furthermore, functional characterization in the heterologous system *Arabidopsis* has confirmed the involvement of KNOX (Zhao et al., 2020), HD-ZIP (Ma et al., 2019), DOF (Ma et al., 2023), and AGL (Wang et al., 2023) transcription factors from litchi in regulating abscission processes. In this study, a total of 15 differentially expressed transcription factors were identified in the AZ (**Figure 5**). Transcriptome analysis revealed complex patterns of differential expression across multiple transcription factor families. Notably, upregulation of specific bHLH (*LITCHI023409*, *LITCHI020266*, *LITCHI022365*, *LITCHI030359*), WRKY (*LITCHI020315*, *LITCHI021320*, *LITCHI028587*), and NAC genes (*LITCHI001198*, *LITCHI022261*), as well as elevated expression of various HSF (*LITCHI008825*), MYB (*LITCHI005562*), RAX (*LITCHI024606*), and TAH (*LITCHI021395*) genes were observed. This diverse array of differentially expressed transcription factors underscores the involvement of intricate transcriptional regulatory networks during preharvest fruit drop. However, it is noteworthy that several transcription factors previously associated with fruitlet abscission were not identified in our analysis of preharvest fruit drop. This discrepancy indicates the existence of potentially distinct regulatory networks for fruitlet abscission and preharvest fruit drop. In summary, this finding not only highlights the intricate interplay between general and specific regulatory elements but also provides valuable insights into the complex molecular frameworks governing the multifaceted character of fruit abscission in litchi.

### 4.4 Reactive oxygen species metabolism in the abscission zone during preharvest fruit drop

Reactive Oxygen Species (ROS) possess strong oxidative properties and serve as signaling molecules that transmit environmental stressors and can trigger programmed cell death. Elevated levels of ROS within cells are known to promote organ abscission, whereas the reduction of ROS can retard this process. The GPD (girlding plus defoliation) treatment of longan bearing-shoots induces fruit abscission concurrently with a surge in ROS levels, and the application of DMTU, a hydrogen peroxide (H_2_O_2_) generation inhibitor, can suppress cellulase activity in the AZ and subsequent fruit abscission (Yang et al., 2015). Upregulation of ROS-related genes has been found in both litchi and citrus fruitlet abscission (Li et al., 2015b; Xie et al., 2018). Aldehyde oxidases, through their antioxidant activities, can regulate ROS levels and are modulated by reactive carbonyl species, which can lead to the overaccumulation of ROS and the onset of oxidative stress (Hasanuzzaman et al., 2020). In this study, we found that one L-aldehyde oxidase gene (*LITCHI021310*) was upregulated in the AZ of “Nuomici”, indicating a potential increase in ROS production in the AZ during preharvest fruit drop (**Figure 6A**). This disparity between ROS generation and scavenging may lead to an oxidative burst within the AZ. In conclusion, the ROS burst observed in the AZ of “Nuomici” litchi is likely a crucial regulatory mechanism underlying preharvest fruit drop.

### 4.5 Cell wall remodeling and programmed cell death in abscission zone facilitate preharvest fruit drop

The separation of cells in the AZ encompasses two key processes: cell enlargement and programmed cell death. Aquaporins play a critical role in accurately regulating the expansion of gap cells (Fricke and Knipfer, 2017). Study on tomato has shown that silencing *SlTIP1;1* can retard the onset of abscission induced by hydrogen peroxide, as reported by Wang et al. (2021). In current investigation, we identified one DEG (*LITCHI029075*) encoding for an aquaporin protein, a member of the plasma membrane intrinsic protein PIP family, that was significantly upregulated in the AZ of “Nuomici” compared to “Huaizhi” during preharvest fruit drop (**Figure 6B**). It suggests an increase in cell turgor pressure within the AZ. Programmed cell death is known to accompany cell apoptosis during the abscission of plant organs, including the shedding of *Delphinium* petals (Yamada et al., 2007) and the self-pruning of sweet orange shoot tips (Zhang et al., 2014), indicating the critical role of PCD in these developmental events. The tomato PIRIN protein, sharing 56% sequence similarity with the human PIRIN ortholog, exhibits a marked increases during the PCD induced by camptothecin in tomato (Orzaez et al., 2001). Here, we observed an upregulated expression of one DEG (*LITCHI015757*), similar to the Pirin-like protein, in the AZ of “Nuomici” (**Figure 6B**), indicating a potential involvement of PCD in preharvest fruit drop process. In summary, preharvest fruit drop in litchi is associated with the enlargement of the AZ and the initiation of PCD processes.

The ultimate event in organ abscission, namely the detachment of the organ from the parent plant, is preceded by the degradation of the cell wall in the abscission zone (AZ). AZ cells expand, applying mechanical pressure that, combined with the weakening effects of cell wall-degrading enzymes, facilitates the rupture of the remaining vascular connections, especially the lignified xylem elements, thereby enabling the complete detachment (Lee, 2019). In this study, two expansin genes (*LITCHI022703*, *LITCHI027667*) were identified as highly expressed in the AZ of “Nuomici” during preharvest fruit drop (**Figure 6C**), suggesting cell wall loosening and cellular expansion. The upregulation of expansin and aquaporins like PIPs indicates intricate molecular mechanisms controlling cell expansion and turgor-driven processes. The degradation of the plant cell wall, a complex matrix of cellulose, hemicelluloses, and lignin, requires orchestrated enzymatic activities for dynamic restructuring during development and in response to physiological cues (Anderson and Kieber, 2020). Specific cell wall-modifying enzymes such as endoglucanases (EG), polygalacturonases (PG), xyloglucan endotransglucosylase/hydrolases (XTH), and pectate lyases (PL) are reported as enzymes involved in cell wall breakdown during the abscission phase (Kim et al., 2019; Zhang et al., 2024). This study shows differential expression of key genes for these enzymes in the AZ, with “Nuomici” displaying an accelerated cell wall degradation compared to “Huaizhi” (**Figure 6C**). The upregulation of two arabinogalactan proteins suggests concurrent synthesis and degradation processes, indicating complex cell wall dynamics during fruit abscission. Additionally, a xyloglucan glycosyltransferase is highly expressed in the AZ of “Nuomici”, potentially contributing to protective layer formation. Two PMEI genes are found to be highly expressed in the AZ of “Nuomici”, suggesting suppressed pectin methylesterase activity, which may be associated with protective layer formation following AZ separation. Overall, the study uncovers a complex interplay of cell wall-modifying enzymes, structural proteins, and regulatory factors orchestrating the cell wall remodeling during preharvest fruit drop in litchi.

## 5 Conclusion

**Figure 9:**
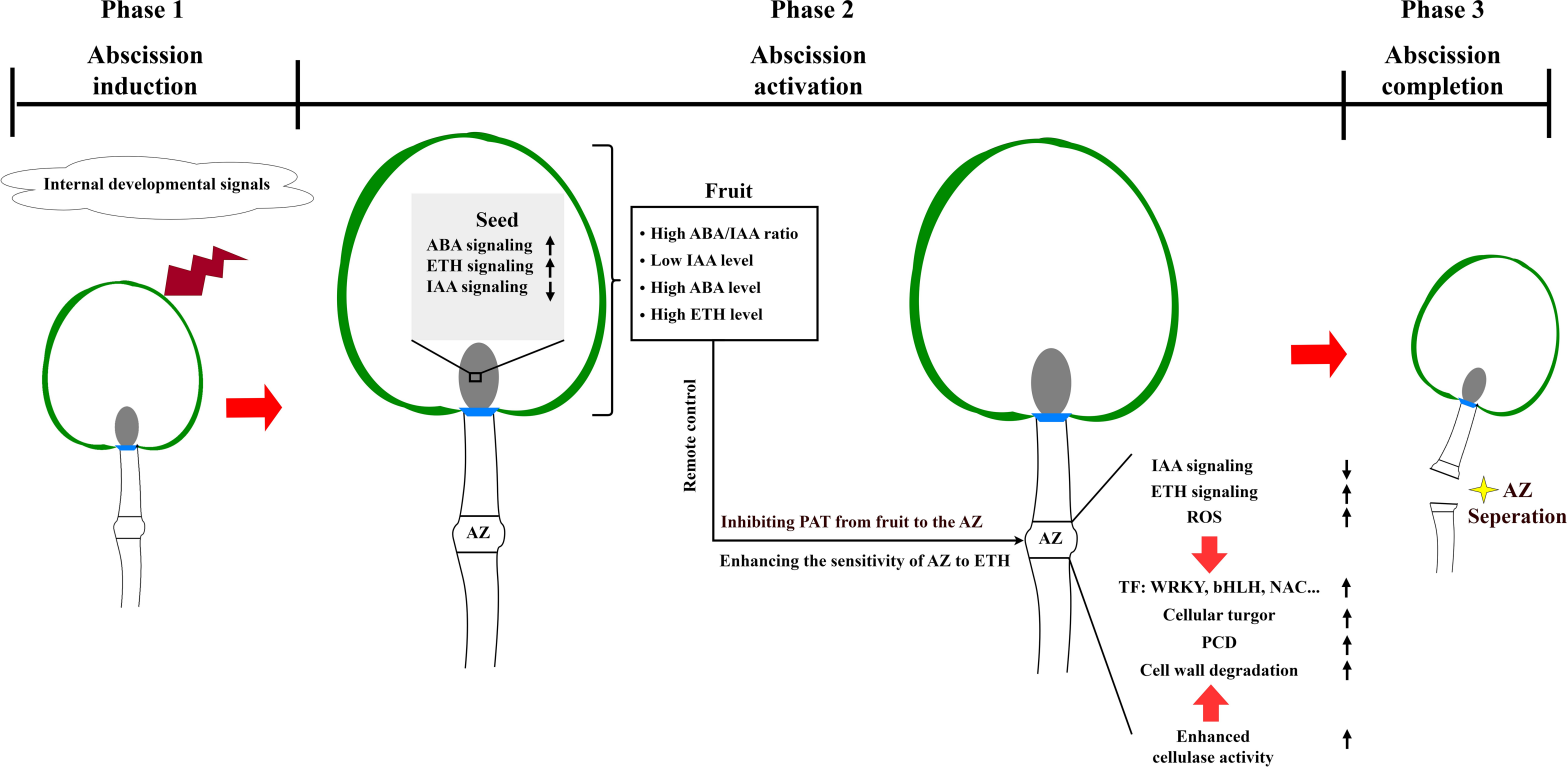
A hypothetical model for preharvest fruit drop triggered by a series of molecular events in litchi. In phase 1, fruits sense the internal developmental signals prior to maturity. In phase 2, the synthesis of hormones and their signal transduction pathways within the seed are altered. This alteration results in the enhancement of abscission signals mediated by abscisic acid (ABA) and ethylene (ETH), while auxin-related signals promoting fruit retention are suppressed. The disruption of hormone balance in the fruit leads to reduced levels of indole-3-acetic acid (IAA), increased levels of ABA and ETH, and variations in other hormonal concentrations. These abscission cues are integrated to inhibit the polar auxin transport (PAT) from the fruit to the abscission zone (AZ), and enhance the sensitivity of AZ to ETH, which in turn activates the abscission process. This activation perturbs hormonal balance further, causing a burst of reactive oxygen species (ROS). The abscission cues exert a “remotely control” by fruit over the AZ, which is involved in cell turgor modulation, programmed cell death, and cell wall degradation. Finally, in phase 3, the cells within the AZ separate, leading to the completion of preharvest fruit drop. The direction of the arrows indicates whether a process is enhanced and decreased, respectively.

Integrating the provided information with insights from abscission models of other organs such as fruitlet abscission (Botton and Ruperti, 2019), leaves abscission (Patharkar and Walker, 2018), and flowers abscission (Meir et al., 2019), a preliminary regulatory model of “remote control” by fruit over the abscission zone (AZ) during preharvest fruit drop in litchi can be postulated. As shown in **Figure 9**, the process begins with the fruit sensing the internal developmental signals before harvest. This triggers modifications in hormone synthesis and signal transduction pathways within the seed, manifested by enhanced abscission signals mediated by abscisic acid (ABA) and ethylene (ETH), and attenuated signals related to auxin. Such hormonal changes affect the fruit’s hormonal balance, leading to the fruit’s initiation of ABA and ETH synthesis while concurrently decreasing the synthesis of growth-promoting hormones, such as indole-3-acetic acid (IAA). Subsequently, the reduced IAA transport from the seed to the AZ disrupts the balance of polar auxin transport in the AZ, heightening its sensitivity to ethylene. Concurrently, ABA may prompt ETH production within the fruit, facilitating its transmission to the AZ through the pedicel and thereby triggering abscission process. These hormonal signals activate distinct transcription factor networks in the AZ, where WRKY, bHLH, and TAF family members coordinate hormone-responsive genes, while NAC, MYB, and HSF transcription factors regulate cell wall modification-related genes. In response to the “remote control” signals from the fruit, the AZ experiences a series of physiological alterations, including a reactive oxygen species burst, and changes in hormone signal transduction, which together elicit the abscission response. This results in cell wall degradation, programmed cell death, and ultimately fruit abscission.

## Data Availability

The data presented in this study are deposited in the National Center for Biotechnology Information (NCBI) BioProject database under accession number PRJNA1140744. Additional related data from our previous work can be accessed under accession number PRJNA681070. The complete datasets are publicly available in the NCBI repository.
