# Supplementary material for: Signaling pathways mediating the induction of preharvest fruit drop in litchi
Source: Front Plant Sci. 2024 Dec 9;15:1474657. doi: 10.3389/fpls.2024.1474657 (PMC11663655; doi:10.3389/fpls.2024.1474657)
Supplement: Supplementary file 1 [file DataSheet1.docx]

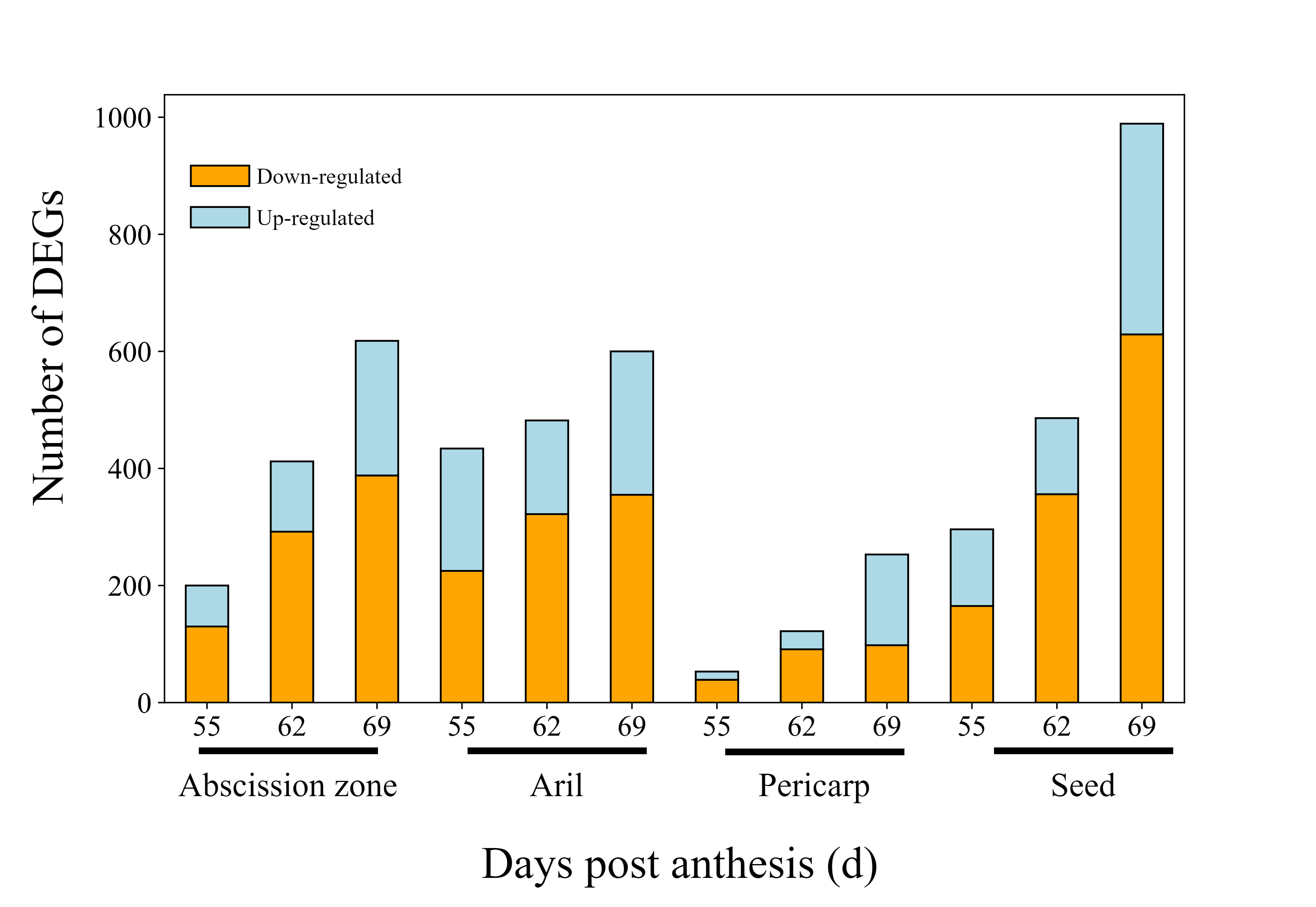


**Figure S1.** Identification of DEGs during preharvest fruit drop in pericarp, aril, seed and abscission zone


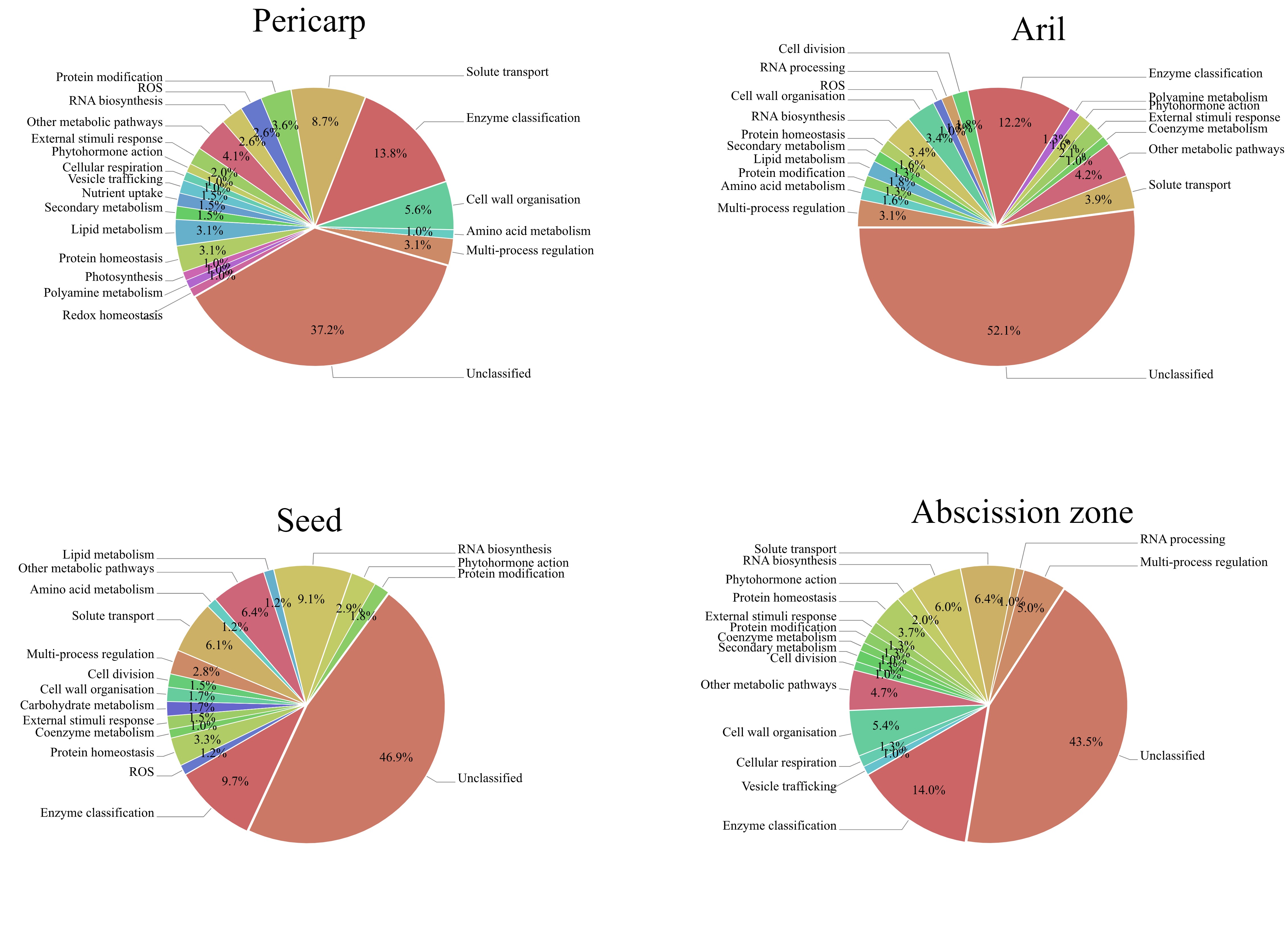


**Figure S2.** Distribution of functional categories of differentially expressed genes in pericarp, aril, seed, and abscission zone tissues during litchi preharvest fruit drop. Functional categories were annotated using Mercator4 in four tissues. Pathways representing less than 1% of total DEGs were combined into "other metabolic pathways".





**Figure S3.** Hierarchical network analysis of enriched GO terms highlighting major biological pathways in litchi abscission zone


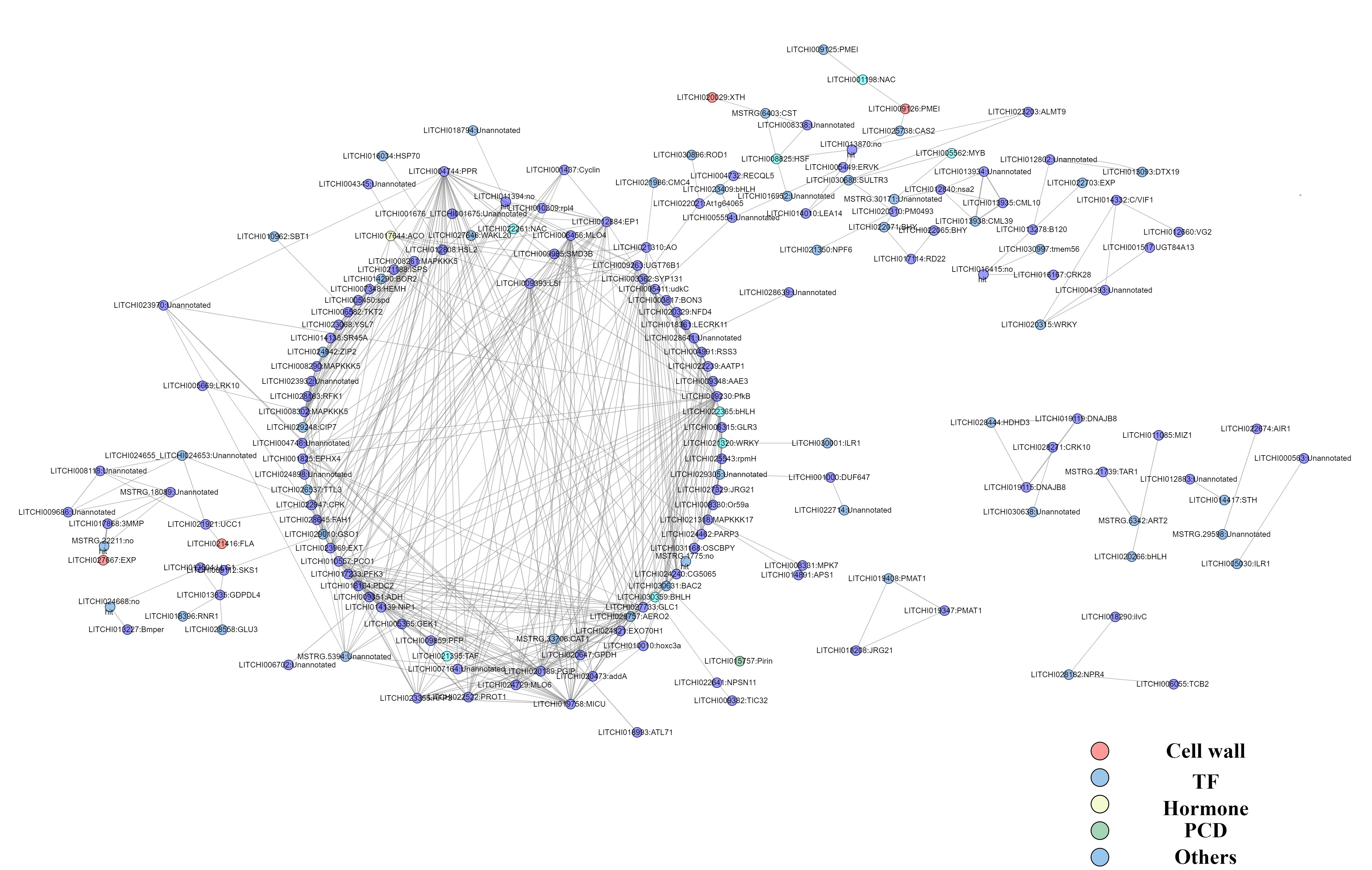


**Figure S4.** Network illustration of co-expression modules associated with preharvest fruit drop in litchi abscission zone
